# Physical characterization of liposomal drug formulations using multi-detector asymmetrical-flow field flow fractionation

**DOI:** 10.1016/j.jconrel.2020.01.049

**Published:** 2020-04-10

**Authors:** J. Parot, F. Caputo, D. Mehn, V.A. Hackley, L. Calzolai

**Affiliations:** a Materials Measurement Science Division, National Institute of Standards and Technology, Gaithersburg, MD 20899-8520, United States; b Theiss Research, La Jolla, California 92037, United States; c Université Grenoble Alpes, CEA, LETI, F-38000 Grenoble, France; d European Commission, Joint Research Centre (JRC), Ispra, Italy

**Keywords:** Field flow fractionation, Liposome, Complex drug, Particle size, Physical-chemical characterization, Method validation, Standardization, Regulatory, AF4, asymmetrical-flow field flow fractionation, DF, channel flow or detector flow rate, DLS, dynamic light scattering, EMA, European Medicines Agency, FBS, fetal bovine serum, FDA, US Food and Drug Administration, FF, focus flow rate, FWHM, full width at half maximum, MALS, multi-angle light scattering, MD, multi detector, NEP, nanotechnology enabled pharmaceutical, NIST, National Institute of Standards and Technology, NNLS, non-negative constrained least squares analysis, PBS, phosphate buffered saline, PI, polydispersity index, PES, polyethersulfone, PSL, polystyrene nanosphere, QELS, quasi-elastic light scattering, RC, regenerated cellulose, *R*_h_, hydrodynamic radius, *R*_g_, root mean square radius, *R*, retention ratio (=*t*_0_/*t*_R_), *R*%, estimated mass recovery, *t*_R_, retention time, *t*_0_, void time, XF, cross flow rate, z-avg, z-average, mean hydrodynamic diameter calculated by cumulants analysis

## Abstract

Liposomal formulations for the treatment of cancer and other diseases are the most common form of nanotechnology enabled pharmaceuticals (NEPs) submitted for market approval and in clinical application today. The accurate characterization of their physical-chemical properties is a key requirement; in particular, size, size distribution, shape, and physical-chemical stability are key among properties that regulators identify as critical quality attributes. Here we develop and validate an optimized method, based on multi-detector asymmetrical-flow field flow fractionation (MD-AF4) to accurately and reproducibly separate liposomal drug formulations into their component populations and to characterize their associated size and size distribution, whether monomodal or polymodal in nature. In addition, the results show that the method is suitable to measure liposomes in the presence of serum proteins and can yield information on the shape and physical stability of the structures. The optimized MD-AF4 based method has been validated across different instrument platforms, three laboratories, and multiple drug formulations following a comprehensive analysis of factors that influence the fractionation process and subsequent physical characterization. Interlaboratory reproducibility and intra-laboratory precision were evaluated, identifying sources of bias and establishing criteria for the acceptance of results. This method meets a documented high priority need in regulatory science as applied to NEPs such as Doxil and can be adapted to the measurement of other NEP forms (such as lipid nanoparticle therapeutics) with some modifications. Overall, this method will help speed up development of NEPS, and facilitate their regulatory review, ultimately leading to faster translation of innovative concepts from the bench to the clinic. Additionally, the approach used in this work (based on international collaboration between leading non-regulatory institutions) can be replicated to address other identified gaps in the analytical characterization of various classes of NEPs. Finally, a plan exists to pursue more extended interlaboratory validation studies to advance this method to a consensus international standard.

## Graphical abstract

**Figure:**
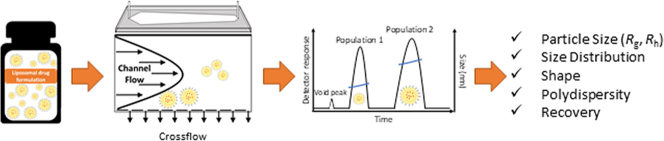

## 1 Introduction

Nanotechnology enabled pharmaceuticals (NEPs) are an emerging class of complex non-biological medical products offering innovative therapeutic and diagnostic opportunities [1]. In particular, liposomal formulations for the treatment of cancer and other diseases are the single most common form of NEP assessed by regulatory agencies for clinical trials and market authorization, both in the US and in the EU, followed closely by nanocrystals. The remaining 36% include 18 different sub-classifications [2]. Despite the initial high expectations for “nanomedicine” to fundamentally alter the way disease is treated [3,4], clinical success has been limited [[5], [6], [7], [8]], in part, due to the failure to fully recognize the complex nature of NEPs and the challenges they present [9,10]. Although progress has been slow, substantial international efforts are now underway in the nanomedicine community to accelerate the translation of promising NEPs to the clinic.

For successful translation into a clinical setting, NEPs must meet the same safety and quality criteria applied to all drug products that do not contain nanomaterials [11]. However, due to their unique and complex nature, the physical-chemical properties and biological profiles of NEPs present substantial analytical challenges relative to small molecule drugs. In general, NEPs must be evaluated using new or modified approaches that address the characteristic heterogeneous and hybrid nature of NEPs without altering the properties of interest. To further complicate matters, each NEP “subclass” is unique and may require different methodologies to evaluate similar critical quality attributes – such as size, physical structure, stability or drug loading. Clearly, a substantial gap now exists between the regulatory need to make informed decisions based on robust and validated characterization methods and the availability of such methods within the nanomedicine community [[12], [13], [14], [15]]. A recent international workshop and companion article on bridging communities in nanomedicine clearly emphasized the urgent need for standardization of methods for regulatory assessment of NEPs [13]. At the moment, there are no internationally recognized standard test methods developed specifically for the physico-chemical characterization of NEPs [15]. The few nanometric standards that do exist are generic in nature (i.e., apply to “nano-objects” or “nanomaterials”), and are not generally suitable for the analysis of complex NEPs [15]. In this context, the development of more accurate and robust characterization strategies to better assess the quality and safety of different subclasses of NEPs is a key requirement for their successful translation into clinical applications. These strategies must ultimately yield robust methods that are validated across instrument platforms, laboratories and product formulations, and which identify significant sources of measurement uncertainty and artifacts.

Importantly, for physico-chemical assessment, a “one size fits all” strategy of measurement standardization has proven unreliable at best, and misleading at worst. Methods must be differentiated for different product classes, due to their unique and variable properties [13]. The US Food and Drug Administration (FDA) and the European Medicines Agency (EMA) have identified liposomal drug formulations as a *high priority* NEP class for measurement advancement and standardization focused on critical quality attributes related to the physico-chemical state [16]. The FDA has published a guidance document for liposomal drugs that includes, among other details, a description of critical physicochemical properties [17]. Moreover, a recent summary on Standards Readiness for Nanomedicine, produced by a study group within International Organization for Standardization (ISO) Technical Committee 229 (Nanotechnologies), identified standardized methods for characterization of liposomal drug formulations as the highest priority need in the nanomedicine field. Thus, the evidence of need is clear and documented.

The present work is focused on assessment of the native physical state of NEPs, including size and size distribution – critical quality attributes relevant to all NEPs. Currently, particle size and sample polydispersity are most often assessed with dynamic light scattering (DLS) performed in batch mode. Data from FDA shows that batch DLS was used to measure particle size in 52% of the NEP applications submitted for evaluation between 2010 and 2015, followed by laser diffraction (30%) and then all other techniques [2]. This extensive dependence on batch mode DLS is problematic because the technique is inherently a low resolution measurement and lacks the capacity to fully resolve the size distribution of multimodal or highly polydisperse and complex samples [13,18]. As a result, reliance on batch DLS analysis can potentially yield misleading results [12,[19], [20], [21]]. Additionally, it is difficult, and often impossible, to measure the physical state and stability of NEP formulations in complex biological media using batch mode DLS. Finally, some of the other common “go-to” methods (e.g., electron microscopy) require substantial sample preparation/modification, such as deposition onto a substrate, cryogenic treatment and/or contrast staining. NEPs such as liposomes are subject to unintended alteration due to the nature of their “soft” structure, and this requires careful selection and application of measurement methodology to avoid changing the analyte as a result of the measurement or sample preparation process.

**Fig. 1:**
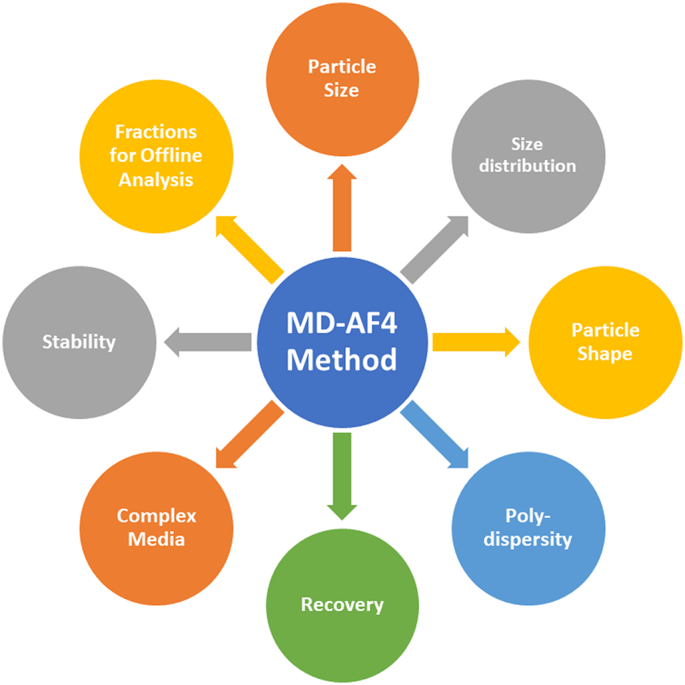
The MD-AF4 method can help both drug developers and regulators alike to better characterize the physical properties of liposomal drug formulations. The method offers much more than simply size analysis, as highlighted in this diagram.

Asymmetrical-flow field flow fractionation (AF4) combined with multiple on-line detectors is a highly effective strategy to overcome the limitations of traditional batch mode DLS, while minimizing sample preparation artifacts. This hyphenated approach increases resolution of size measurements, enables analysis of complex multimodal samples and is generally compatible with complex media [22]. Multi-detector (MD) AF4 was therefore chosen for the present study due to its flexibility and broad application to biomacromolecules [23] and biomedical nanomaterials [24,25], including lipid based systems, such as liposomes [[26], [27], [28], [29], [30], [31], [32], [33]], lipid nanoparticles [20] and extracellular vesicles [34,35], among others. In particular, MD-AF4 has the capacity to yield a rich physical dataset on NEPs, as schematically illustrated in Fig. 1.

**Fig. 2:**
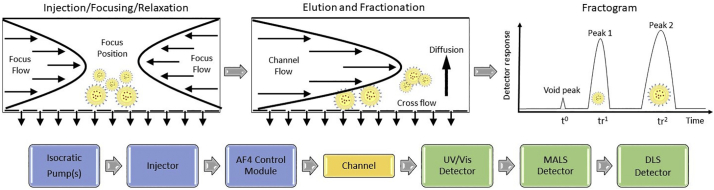
Top from left to right, schematic illustration of injection/focusing/relaxation step, elution with fractionation, and resulting fractogram for AF4. Bottom left to right, simplified flow diagram showing principal components of MD-AF4 instrument systems used in this study*.*

Commercially available AF4 systems can be coupled with a wide range of on-line detectors to provide tandem measurement of size, molar mass, mass concentration, shape factor and, for many elements, composition. Fractionation occurs in the mobile liquid phase as it flows through a thin channel in which analyte diffusion is counterbalanced by a downward force provided by cross flow through an underlying porous membrane (referred to as the accumulation wall). This process results in differential particle velocities across the channel based primarily on analyte size, where smaller particles exit the channel before larger ones, and particle populations form separate bands that are detected and quantified, as represented in Fig. 2. Because the particles are separated according to size, individual fractions or slices in time are essentially monodisperse, mitigating the deconvolution issue associated with batch mode DLS (and other ensemble scattering techniques).

The principal challenge for MD-AF4 is the need to develop and validate individualized methods for different NEP subclasses and, in some cases, specific formulations or even specific applications. The current work builds upon an existing ISO Technical Specification that defines the general application of AF4 to the analysis of nano-objects (ISO/TS 21362). The objective was to establish a validated best practice method based on MD-AF4 for the physical analysis (e.g., size and size distribution, modality, shape, etc.) of nanometric liposomal drug formulations.

The prototype selected to develop and optimize the MD-AF4 analytical methodology for liposomal drugs is a research grade, monomodal, doxorubicin HCl liposome and its (drug-free) control (herein designated as Dox1 and Dox1C) [36]. The prototype possesses physico-chemical properties identical to that of the reference listed drug Doxil® (distributed as Caelyx® in the EU) [37].^1^ This reference listed drug was approved by the FDA in 1995 and EMA in 1996, and has been used to treat over 600,000 cancer patients world-wide as of 2016 [38]. The formulation consists of the chemotherapy drug doxorubicin HCl crystallized within the internal aqueous cavity with ammonium sulfate in a spherical unilamellar lipid bilayer structure composed of hydrogenated soybean phosphatidylcholine, cholesterol and methoxy-polyethylene glycol (2000)-1,2-distearoyl-sn-glycero-3-phosphoethanolamine (PEG2000-DSPE). The last component is integrated with the external lipid layer providing steric stabilization and prolonged circulation by evasion of the macrophage system. The liposomes are suspended in an aqueous solution containing histidine buffer and sucrose to maintain isotonicity.

A systematic investigation was conducted with Dox1 to assess potential factors impacting the quality and reproducibility of liposome fractionation and the determination of liposome size and physical state. Based on these extensive measurements, a basic method was established and validated across different instrument platforms, laboratories and formulations. This method was then challenged using a research-grade polydisperse liposomal doxorubicin HCl product (Dox2), presenting a more complex physical state relative to Dox1. The method was further validated using two FDA-approved Doxil generics identified herein as Dox3 and Dox4. Measurement parameters were sufficiently flexible to accommodate both monodisperse and polydisperse test samples, while yielding optimal analytical parameters such as selectivity, recovery and reproducibility. The method was then successfully applied to a research-grade PEGylated liposomal ciprofloxacin (an antibiotic drug), denoted here as Cipro. Finally, the study demonstrates that the method is suitable to measure the physical properties of liposomes in complex protein-containing biological media such as plasma.

The study also identifies sources of bias and poor performance, and criteria for acceptance of data were established. We briefly address issues related to the accuracy and reliability of particle size measurements based on multi-angle light scattering (MALS) and DLS in flow mode on different instrument platforms; this topic will be more fully explored in a subsequent publication. Critical points and good practices are identified to aid the implementation of the method.

Finally, the optimized method developed and validated in this study provides a structured yet flexible approach that can be employed across current commercial instrument platforms and applied to most if not all nanometric liposomal drug formulations. The method should be appropriate for purposes of pre-clinical research/development, product quality control and regulatory assessment. The intent is that this method will form the basis for development of an international consensus standard that expedites the regulatory review process and supports the development and broad adoption of NEPs in the treatment and detection of disease.

## 2 Experimental

### 2.1 Materials

Polystyrene (PSL) 3000 series nanosphere NIST Traceable® size standards, with nominal diameters of (30, 60, 125, 200 and 350) nm, were purchased from ThermoFisher Scientific (Waltham, MA USA). Bovine serum albumin (BSA) monomer (> 99%), blue dextran salt and fetal bovine serum (FBS) were purchased from Sigma Aldrich (St. Louis, MO USA).

Phosphate buffered saline (PBS) used as a mobile phase in the experiments was purchased from ThermoFisher Scientific (catalog #18912), GE Healthcare Life Sciences (Pittsburgh, PA USA, catalog # 16777–252) and Lonza (Basel Switzerland, PBS catalog #17-516Q and DPBS catalog #17-512Q). Sodium chloride was purchased from Sigma Aldrich. Table S1 describes in detail the chemical composition of the mobile phase used in different experiments. Prior to measurement, the mobile phase was filtered through 0.1 μm Pall Acrodisc Supor Membrane filters obtained from VWR Scientific (Philadelphia, PA USA). Regenerated cellulose (RC) and polyethersulfone (PES) channel membranes and other channel components were purchased from Wyatt Technology (Santa Barbara, CA, USA) and Postnova Analytics GmbH (Landsberg am Lech, Germany).

The properties of the liposomal samples used in this study and the coding used to identify them in the text are summarized in Table S2. The Dox1 formulation (batch #101071) and its drug-free control Dox1C (batch #500010) were purchased from Lipocure Ltd. (Jerusalem, Israel). Dox1 is sold under the name DoxoCure and its physical-chemical parameters are identical to the reference listed drug Doxil® [37]. The Dox2 sample (product code 300115, lot #300115–01-010) and its control Dox2C (product code 300116, lot #300116–01-010) were purchased from Avanti Polar Lipids (Alabaster, AL USA), and are stored frozen in 10% sucrose and 10 mmol/L histidine buffer. Dox2 is sold under the product name DOX-NP®, but should not be confused with the previous product sold under the same tradename but manufactured by Lipocure Ltd. until 2017. FDA-approved Doxil® generics were obtained through a research pharmacy. Dox3 (NDC 43598–283-35) is distributed by Dr. Reddy's Laboratories Inc. (Princeton, NJ USA) and Dox4 (NDC 47335–050-40) is distributed by Sun Pharmaceutical Industries Inc. (Cranbury, NJ USA). Research grade PEGylated hydrogenated soybean phosphatidylcholine liposomal ciprofloxacin (product code PHPC002CP), denoted here as Cipro, was obtained from ProFoldin (Hudson, MA USA).

### 2.2 Sample preparation

Prior to measurement, each sample (stock suspension) was diluted into the mobile phase at a lipid concentration of 1 mg/mL and stored refrigerated at (2–4) °C until needed. Dox2 was aliquoted and stored frozen at −20 °C (according to manufacturer directions); aliquots were allowed to thaw before use and stored refrigerated after dilution. To study the suitability of the method to analyze liposomal drugs in complex biological media, samples of Dox1 and Dox2 were diluted in PBS + 10% volume fraction fetal bovine serum (FBS) immediately before injection. No further sample preparation was required for MD-AF4 analysis.

*Best Practice Note – To maintain sample integrity, stock suspensions should be refrigerated until diluted for MD-AF4 analysis. Where high throughput analysis is required, a thermostatted autosampler is recommended.*

### 2.3 Batch DLS measurements

Batch mode DLS measurements were performed at 25 °C using a Zetasizer Nano ZS (Malvern Panalytical, Westborough, MA USA) equipped with a 633 nm laser and operating in backscatter detection. Prior to measurements all samples were diluted in PBS at 1 mg/mL (total lipid concentration). Size results were obtained by averaging 5 consecutive measurements. The results of cumulants analysis, the mean hydrodynamic size (*Z-avg*) and polydispersity index (*PI*), are reported. Intensity-weighted hydrodynamic size distributions generated by non-negative constrained least squares (NNLS) analysis are also reported where appropriate.

### 2.4 MD-AF4 measurements

#### 2.4.1 Instrumentation

Three widely available commercial platforms were utilized for this study. Included were an Eclipse DualTec (Wyatt Technology), an Eclipse AF4 (Wyatt Technology) and an AF2000 Multiflow FFF (Postnova Analytics). All platforms included necessary isocratic pump(s), degasser, injector, and fractionation channels. Additionally, each system was equipped with a minimum of three online detectors relevant to the present work: MALS, UV–Vis absorbance and DLS. For the AF2000, the DLS detector was a Zetasizer Nano ZS (Malvern Panalytical) operating in flow-mode, while for the Wyatt systems the DLS detector was integrated into the MALS. The specifications for each detector vary somewhat (e.g., angular range, number of angles for MALS), but for the purpose of the present work, all detectors were capable of performing the necessary measurements for optimization and application of methodology. For a detailed description of each system, including detectors, refer to Supplemental Information (SI) Section 1. MALS angles for each system are shown in Table S3.

#### 2.4.2 Fractionation

Fractograms were obtained by injecting an appropriate volume of sample into the AF4 system (typically of order 25 μL). The mobile phase (eluent) is pumped into the channel from opposite ends during injection and focusing, and exits through a replaceable semi-porous membrane at the accumulation wall (Fig. 2). When focusing is complete the analyte forms a thin line transverse to the direction of channel (and detector) flow. Subsequently the elution program is initiated, and the analyte is separated into components based on size as a result of opposing forces of particle diffusion and an applied cross flow rate (XF) perpendicular to channel or detector flow rate (DF)^2^ (Fig. 2). The void peak elutes first, followed by analyte components in order of increasing size. The eluting analyte components move toward the exit port of the channel and then on to the tandem detectors. Injection, focusing, XF, DF, elution program, injected mass, membrane type and molecular weight cut-off (MWCO), mobile phase composition, channel height and length and conditioning/washing steps are optimized during method development.

The basic fractogram consists of elution time on the abscissa and detector response(s) on the ordinate axis (Fig. 2). Here *t* = 0 is the initiation of analyte elution with cross flow, following sample injection and focusing steps. The void time, t^0^, is identified from the eluting void peak shown in Fig. 2, which contains any unretained material and travels at the mean velocity of the DF. From the fractogram, analyte peaks or fractions are identified by their retention time, t_R_, which is generally assigned at the peak maximum, and their retention ratio, R = t_0_/t_R_, which normalizes results to the void peak. The UV–Vis trace (mass detector) was used to define retention and void times. Unless stated otherwise, all flow rates, including injection, focus, channel/detector and cross, are given in units of mL/min (e.g., a channel flow of 0.5 mL/min is shown as DF = 0.5).

#### 2.4.3 Separation efficiency, selectivity and quality

The efficiency of a separation was assessed as a balance between the speed of analysis and the resulting resolution, while allowing multiple populations possessing different physical properties (i.e., size and shape) to be resolved if present. If relevant size standards were available, one could also assess selectivity (change in size relative to a change in retention time), but currently there are no available size standards that are suitable for the present application (i.e., for liposomes in PBS). We assessed the general quality of the fractionation by considering the shape and efficiency of the eluting peak(s), and the retention ratio (where lower values indicate increased separation from the void peak).

#### 2.4.4 Online size measurement

The online MALS detectors were calibrated at a scattering angle of 90° and the remaining detector angles normalized to the response at 90° using an isotropic scatterer (e.g., BSA in PBS) according to manufacturer recommendations. This process yields absolute scattering intensity (Rayleigh ratio, cm^−^^1^) and ensures that all detector angles perform equally. The excess Rayleigh ratio is determined for fractionated samples (i.e., scattering from the pure mobile phase is subtracted from the sample signal). The resulting absolute intensity at 90° is typically presented in a self-normalized manner. For each “slice” or data point in a fractogram, the excess Rayleigh ratio is analyzed versus scattering angle and fit with an appropriate scattering equation (e.g., Berry form of the Debye model in the case of liposomes or the sphere form factor in the case of PSL) [39,40]. The number of angles and angular range varies for different detectors. Data points are selected based on the quality of the fit, and the output is the root mean square radius (commonly referred to as the radius of gyration), *R*_g_, where $R_{g}=\sqrt{3/5}R_{s}$ and *R*_s_ is the geometric radius of a solid sphere.

*Best Practice Note: Calibration and normalization should be performed at least once per year and anytime the MALS flow cell has been disassembled (*e.g.*, for cleaning). Normalization of all detectors to 90° should be confirmed, and, if necessary, corrected each time the mobile phase composition is changed.*

Online DLS measurements were performed using either a Wyatt QELS (Quasi-Elastic Light Scattering) integrated directly with the MALS at an angle of 99.9° or 134° or using a Malvern Zetasizer positioned as the last detector and operating in flow-mode with backscatter detection at 173°. The measured correlation functions obtained during fractionation were analyzed using either single exponential decay or the cumulants method, both of which should theoretically yield the same value at the same angle if the eluting sample is size-fractionated (i.e., monodisperse). The output is the equivalent sphere hydrodynamic radius, *R*_h_.

Where appropriate, *R*_g_ and *R*_h_ are reported on the fractogram across the full width at half maximum (FWHM) for fractionated peaks that are adequately defined, or near the peak or shoulder maximum if ill defined. In tabulated results, *R*_g_ and *R*_h_ are averaged across the FWHM (for monomodal samples) or reported at peak maxima (for polymodal samples). The spread of size values across the FWHM is reported as a measure of peak polydispersity (where spread = difference between the minimum and maximum size values across the FWHM). To perform these measurements and to present data in a fractogram format, scattering intensity at 90° is used for *R*_g_ and *R*_h_ (measured by QELS in the MALS flow cell), whereas for *R*_h_ measured using a Zetasizer in flow-mode, the detector count rate (unattenuated) at the angle of measurement (173°) is used instead. Finally, the Burchard-Stockmayer shape factor is calculated from the ratio of the root mean square radius and the hydrodynamic radius (*ρ* = *R*_*g*_/*R*_*h*_), where ρ = 0.775 for a solid sphere and 1 for a thin hollow sphere [41].

#### 2.4.5 Mass detection and analyte recovery

Online UV–Vis absorbance at 280 nm was utilized for mass detection during fractionation and for determination of the analyte mass recovery. Mass recovery, *R%*, was estimated by integrating the area under the UV–Vis peak for each sample eluted with and without the applied XF and focusing step, as follows:

(1) $$R\%=\left(\frac{\mathrm{UV}-\mathrm{Vis}\;\mathrm{area of fractionated analyte}}{\mathrm{UV}-\mathrm{Vis}\;\mathrm{area of analyte without crossflow or focus}}\right)*100$$

For AF4, recovery is considered acceptable if analyte loss is ≤30% of the total injected mass [42]. In the liposomal formulations tested in this study, an unfractionated but retained component elutes immediately after XF is removed. For present purposes, this material is included in the recovery determination (i.e., in the numerator of Eq. 1).

*Explication – The unfractionated-retained peak contains material that is retained but not size separated, and which elutes only after cross flow ceases. This peak might contain, for instance, a mixture of co-eluting large structures.*

*Critical Point – Recovery calculations should include the unfractionated-retained peak, since technically it is not “lost” during the analytical process and is a component of the original sample.*

*Critical Point – Depending on the absorbance properties of the active pharmaceutical ingredient, a wavelength specific to this component can be used for recovery estimation or to differentiate between free and encapsulated drug. Additionally, other mass detectors could be used depending on the specific properties of the analytes (*e.g.*, fluorescence, refractivity).*

#### 2.4.6 AF4 performance verification

Following the instrument manufacturer's procedure, the analyte focus position was adjusted as necessary prior to measurements. A position between (10 and 15) % of the channel length is recommended.

Prior to starting a series of measurements or after replacement of the channel membrane or following any significant alteration or maintenance on the AF4 system, (50–150) μg of BSA in PBS mobile phase was injected as a quality control material to verify performance, account for detector delays and correct for band broadening. Any unusual or unexpected fractionation results observed for BSA triggered an investigation of system integrity. BSA could also contribute to membrane passivation, but this is not the primary intent of this step and we do not believe passivation is necessary in the application of this method.

The performance and acceptable upper size range for online MALS and DLS (Wyatt QELS, Malvern Zetasizer) detectors was confirmed using NIST Traceable® PSL sphere size standards from 15 nm to 175 nm (radius) with a coefficient of variation <3% (AF4 PSL method in SI Section 2).

#### 2.4.7 Uncertainty and precision

Due to the complexity, modular nature and variations in commercial platforms for MD-AF4, determination of uncertainty for multiple measurands and for the fractionation process itself are difficult to establish. For online size measurement using MALS and DLS, various models and fitting procedures are available to the user, but the calculation of underlying error is not always transparent. Uncertainty for size endpoints can be estimated by using available PSL standards, though measurements in this case are not conducted under identical conditions to the analyte. This is an especially critical issue going forward, given the increasing use of AF4-based analytical methods. On the other hand, precision (repeatability of the measurement or process) is fairly straight forward in this case. In the present study, precision was evaluated for different endpoints as the standard deviation of the mean calculated from at least 3 replicate measurements (fractionations). Replicates are considered acceptable if the calculated standard deviation is <5% of the mean value, as recommended in ISO TS 21362 [42].

#### 2.4.8 MD-AF4 optimization

Method optimization was performed following the general approach outlined in Gigault et al. [43] and using guidelines specified in ISO TS 21362 [42]. In the first step, different elution profiles were evaluated over a range of XF/DF conditions, including constant XF, linear XF decay and exponential XF decay. Using a constant XF elution program, multiple key AF4 parameters were evaluated, including (i) short versus long trapezoidal channels (ii) membrane type and MWCO, (iii) channel (spacer) height, (iv) DF and XF, (v) focus + injection/relaxation time and focus flow rate (FF), (vi) injected analyte mass and (vii) mobile phase composition as described in Table S1 (including PBS, Dulbecco's PBS (DPBS) and isotonic saline). Additionally, other procedures were tested or varied to identify optimal conditions with respect to fractionation quality, repeatability, and recovery. For instance, memory effects were assessed and addressed by incorporating a mobile phase injection and elution procedure (sans analyte) between sample runs (infra vide).

*Best Practice Note – Channel pressure should be monitored during the different steps of the focusing and elution program to ensure it remains within the manufacturer's acceptable range. Substantial or unexpected changes in pressure can indicate problems with the membrane or the flow path.*

For details regarding data analysis and reporting for this method, including presentation of fractograms and assignment of retention times, refer to Supplemental Information Section 2.

## 3 Results and discussion

### 3.1 Preliminary screening by batch mode DLS

**Fig. 3:**
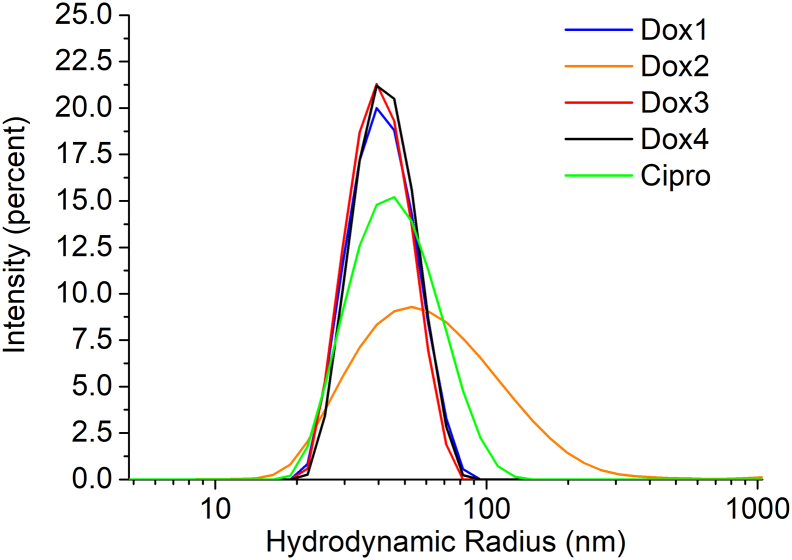
Batch mode DLS: Representative intensity-weighted particle size distributions (R_h_) of Dox1 (Blue), Dox2 (orange), Dox3 (red), Dox4 (black) and Cipro (green) obtained from NNLS analysis of measured correlation data. (For interpretation of the references to colour in this figure legend, the reader is referred to the web version of this article.)

As an initial check of sample size and polydispersity following standard industry practise, batch mode DLS measurements were performed on all liposomal samples used in the present study. Detailed results obtained from cumulants and NNLS analysis are summarized in Table S4. Dox1, Dox3 and Dox4 yielded a *Z*-avg radius of 40 nm and a PI <0.06, indicative of monomodal and relatively monodisperse samples. This conclusion is also confirmed by the intensity-based particle size distribution (PSD) analysis reported in Fig. 3. Dox2, on the other hand, is characterized by a PSD shifted to larger size values and a significantly higher PI (0.26), indicative of a sample with moderate to substantial polydispersity. Moreover, the size values for Dox2 obtained by the cumulants analysis (*Z*-avg *R*_h_ = 56 nm) and by the intensity-weighted NNLS analysis (principal peak mean radius: 73.5 nm) differ by about 30%. This suggests the presence of multiple populations within Dox2 that batch mode DLS, due to its low resolution, is unable to resolve. Cipro yielded a Z-avg radius of 43.5 nm and a PI of 0.01 (Table S4), again indicating a monomodal and relatively monodisperse formulation.

Batch mode DLS is not always an appropriate method to measure the mean size and PSD of NEPs, unless orthogonal data exists to support its use and interpretation. For instance, it is widely recognized that if the sample is polydisperse and/or multimodal in nature, the resolving power of batch DLS is greatly limited and can yield misleading results or limit the ability to identify significant differences between similar test samples. On the other hand, MD-AF4 offers greater capacity to resolve multiple components and to “fingerprint” the physical state of complex formulations. To obtain a more accurate PSD for these challenging materials, MD-AF4 provides substantial advantages, as discussed previously. The relevant experimental parameters were fine tuned to obtain an optimized MD-AF4 method.

*Best Practice Note – Samples should be pre-screened using batch DLS to quickly provide estimates of size and polydispersity.*

### 3.2 MD-AF4 method optimization

#### 3.2.1 Elution program (application of cross flow)

Elution programs can be fine-tuned to improve efficiency of fractionation and speed of analysis, particularly when the sample is multimodal. Essentially, XF is varied during the elution step according to a predetermined program, where the simplest version applies a constant XF rate throughout the fractionation process. Multiple elution programs were evaluated to determine the optimal approach and to assess to what degree analysis results are impacted by the choice of program. Unless otherwise stated, the following conditions were applied during optimization of the elution program and cross flow: PBS mobile phase, long channel, 350 μm spacer, 10 kDa RC membrane, DF = 0.5, FF = 2 and 50 μg injected mass.

**Fig. 4:**
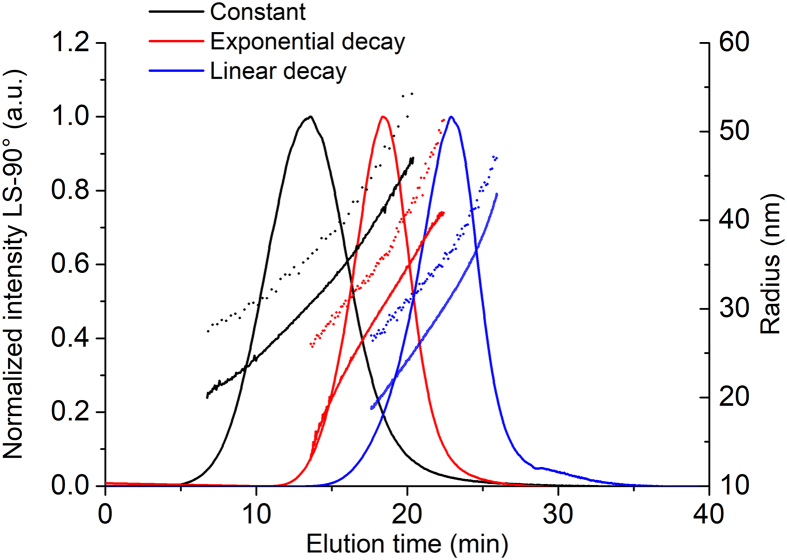
Comparison of elution programs applied to Dox1. AF4 fractograms resulting from different elution programs (normalized excess Rayleigh ratio at 90°), R_g_ (line) and the R_h_ (dots) as a function of elution time for injection of 25 μg of Dox1. Constant XF = 0.3 (black), exponential decay from XF = 1.0 to 0 in 60 min (red) and linear decay from XF = 1.0 to 0 in 30 min (blue). (For interpretation of the references to colour in this figure legend, the reader is referred to the web version of this article.)

The fractograms for Dox1 are compared in Fig. 4 and in Figs. S1-S2, while the mass recovery (*R*%) measured at 280 nm, *t*_R_, *R*, the mean and spread of *R*_g_ and *R*_h_ are reported in Tables S5-S8. Mass recovery was always >90%, for all programs tested. However, different programs yielded different fractogram profiles, with the maximum elution time at 13.53 min for constant XF at 0.3, 19.29 min for the exponential decay from XF = 1.0 to 0 in 60 min, and 23.36 min for the linear decay from XF = 1.0 to 0 in 30 min.

On the other hand, the size profiles and the quality of the separation for almost all programs tested are remarkably similar for Dox1; that is, the data indicates a single population with a mean *R*_g_ of about 30 nm, a mean *R*_h_ of about 35 nm, a size spread (max-min value at the FWHM) of about 10 nm, and a Burchard-Stockmayer shape factor (ρ = *R*_g_/*R*_h_) of 0.84. The shape factor is intermediate between the theoretical value for a solid sphere (0.77) and that for a thin hollow sphere (1.0), and is therefore consistent with the known structure of doxorubicin HCl encapsulated liposomes (i.e., a core shell structure where the core is not empty, but not uniformly solid either). The difference might also be within the statistical uncertainty of this ratio determination. The only exception here was the long linear decay (45 min or longer), which resulted in broad asymmetric peaks (see SI, Fig. S1B and Fig. S1C). Even if the efficiency of separation of the analyzed monomodal sample is satisfactory for all profiles tested, the constant XF profile might have better selectivity and resolution when analyzing multimodal samples (though potentially at the expense of speed of analysis). For this reason, and also because of its simplicity in method development across different instrument platforms and software packages, the constant XF program was selected for further method optimization, with XF = 0.3. The XF value was selected based on a comparison of fractionation results (data not shown) obtained for DF = 0.5 and higher; this XF value generally worked well for all liposomal formulations tested.

#### 3.2.2 Detector flow rate and determination of upper size limits

DF was optimized by evaluating its effect on the retention time, separation selectivity and recovery. Unless otherwise stated, the following conditions were applied during optimization of DF: PBS mobile phase, short channel, 350 μm spacer, 10 kDa RC membrane, XF = 0.3, FF = 2 and 25 μg injected mass. Varying DF from 0.5 to 1 did not significantly impact the separation selectivity or analyte recovery, though it does impact the peak breadth somewhat (see Fig. 6F). Importantly, its influence on the upper size limit measurable by the online DLS detectors was considered. It is known that DF can impact the upper size measurable by online DLS detectors [44], while MALS is much less sensitive. In flow mode, the slower Brownian motion (and longer correlation decay time) of larger particles requires a longer residence time in the measurement zone to be accurately analyzed by DLS. The larger the particle the longer the correlation decay. For this reason, online DLS measurements tend to underestimate the *R*_h_ values, and the systematic error in the *R*_h_ measurements is larger, when higher DF rates are applied and/or larger particles are measured.

**Fig. 5:**
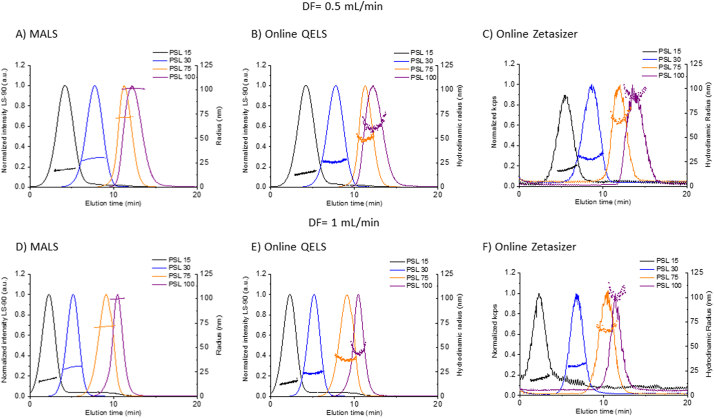
Determination of the upper size limits using online detectors. AF4 fractograms (normalized excess Rayleigh ratio at 90°) for PSL (15–100) nm (radius) with R_h_ at FWHM (dots) overlaid. (Top) A) MALS (Wyatt), B) online QELS and C) online Zetasizer (Malvern) measured at DF = 0.5. (Bottom) radius measured at DF = 1.0 by D) MALS, E) online QELS and F) online Zetasizer.

In order to assess the suitability of the online size detectors used in typical instrumental setups in the size range relevant for most liposomal formulations (i.e., radius from about 15 nm to about 100 nm) we first measured PSL size standards of nominally (15, 30, 62.5, 75, and 100) nm radius by applying two different flow rates: DF = 0.5 and 1, which covers the typical range of use for AF4. The performance of the MALS detector, the QELS detector at two angle positions (99.9° and 134°) and the Zetasizer (in flow-mode) were analyzed. The fractograms are plotted in Fig. 5, while the mean size values calculated across the FWHM are summarized in Table S9. Size calculated by MALS using the sphere form factor (i.e., the sphere model) was always consistent with the stated size value, and constant across the eluting peak (as expected for single mode monodisperse populations). In contrast, the QELS position/angle directly affects the *R*_h_ values with better accuracy at position 16 (scattering angle 134°) in comparison with position 12 (scattering angle 99.9°) for particles larger than 75 nm (radius). Using position 12, *R*_h_ values for PSL ≥ 75 nm were underestimated, while at position 16 results were acceptable up to 175 nm (Table S9). This effect is well known to the instrument manufacturer, who has developed a larger bore cell for applications involving larger particles (> 75 nm) independently of the QELS position. The manufacturer also recommends installing the QELS detector at a higher angle (e.g., position 16) in the standard MALS flow cell for larger size particles (e.g., *R*_h_ > 75 nm). Consequently, the two QELS positions (12 and 16) can be used to measure the size range relevant for most liposomal drug formulations. Therefore, we conclude that the upper size limit will be slightly reduced at DF = 1 compared to 0.5. However, even at DF = 0.5, in our configuration, the upper size limit for DLS using the Wyatt QELS integrated detector with the standard flow cell is about 75 nm (radius), while no upper size limit was observed for the MALS detector (*R*_g_) within the diameter range evaluated. The vendor (Wyatt) states in their literature that the applicable *R*_g_ range for the configuration and laser wavelength used in this study is from about (10 to 250) nm. Therefore, when using the Wyatt MALS/QELS combined detector with the standard flow cell, it is recommended that the analyst rely on *R*_g_ alone for size when the measured *R*_h_ is larger than about 75 nm. For the Zetasizer DLS operating in flow-mode as the final tandem detector, no upper size limit was observed using PSLs up to 100 nm in radius, at DF = 0.5 or 1. However, to obtain acceptable *R*_h_ results from the Zetasizer in flow-mode, it might be necessary to increase the injected analyte mass relative to that used with the online QELS. This depends strongly on the size and scattering properties of the analyte.

*Critical Point – The angle of DLS measurement can significantly affect the measured R*_h_*. This effect becomes more noticeable as particle size increases.*

*Best Practice Note – The upper size limit for R*_h_*measurements on a given instrument platform should be confirmed using size standards over a range that is relevant for the target analyte.*

Notably, for Dox1, which is below the upper *R*_h_ limits established in this study, the *R*_h_ values calculated at higher DF were nevertheless slightly smaller compared with lower DF, even though separation selectivity and recovery results were acceptable at either flow rate (see Table S10 and Fig. S3). In order to minimize potential bias induced by DF on the measured *R*_h_ values we selected DF = 0.5 for further optimization, as it offered a larger operational range and consistent *R*_h_ values.

#### 3.2.3 Channel and membrane properties

**Fig. 6:**
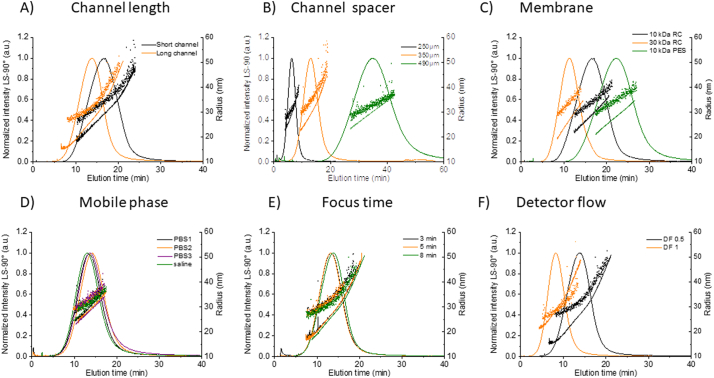
Method optimization results for Dox1*.* Fractograms showing the elution trace (normalized excess Rayleigh ratio at 90°), R_g_ (line) and R_h_ (dots) versus elution time for injection of 25 μg Dox1, constant XF = 0.3 and varying A) channel length (black: short channel, orange: long channel, B) channel spacer (black: 250 μm, orange: 350 μm, green:490 μm), C) membrane (black: RC 10 kDa, orange RC 30 kDa, green: PES 10 kDa) D) mobile phase (green: isotonic saline = NaCl 0.9%, black: PBS from Lonza (PBS1), orange: DPBS from Lonza (PBS2), purple: PBS from Hyclone (PBS3), E) focus time (black: 3 min, orange: 5 min, green: 8 min) and F) detector flow (violet: 0.5 mL/min, green: 1.0 mL/min) are reported. (For interpretation of the references to colour in this figure legend, the reader is referred to the web version of this article.)

The influence of key channel parameters (i.e., channel length, spacer thickness and membrane type/MWCO) on the fractionation process was evaluated for Dox1. Unless otherwise stated, the following conditions were applied during optimization of the channel and membrane properties: PBS mobile phase, DF = 0.5, FF = 2, XF = 0.3 and 25 μg injected mass. Channel length does not affect separation selectivity, mass recovery or the measured size values (see Fig. 6A and in Table S11). Therefore, both the standard “long” (275 mm) and “short” (145 mm) channels from Wyatt can be applied in the method with similar results. The benefit to using the shorter channel is a reduction in analysis time and potentially some reduction in band broadening (though significant band broadening was not observed with Dox1), while the long channel more closely matches the standard channel length (280 mm) used on the Postnova platform. All channels tested use the standard trapezoidal geometry (where the channel breadth decreases toward the outlet), as it regulates the channel flow velocity and minimizes band broadening [45].

Three spacers were evaluated to establish the effect of channel height, which directly impacts the parabolic velocity profile (see Fig. 2) between the accumulation wall (membrane surface) and the solid channel top called the depletion wall; viz. 250 μm, 350 μm and 490 μm (see Fig. 6B and Table S12). Based on these tests, the 350 μm spacer was selected as the best compromise to minimize peak broadening and retention time, while achieving good detector sensitivity (a function of sample loading and dilution) and retention ratio (separation from the void peak).

Finally, we assessed the two most commonly used and widely available membrane materials for AF4; viz. RC and PES. For this comparison, membranes with 10 kDa MWCO were used for fractionation of Dox1. As summarized in Table S13 and shown in Fig. 6C, the PES membrane increased analyte loss compared to RC, though the mass recovery was above 70% (and thus acceptable) for both. In addition, the PES membrane resulted in increased retention time and peak broadening, indicating there is greater interaction between PES and the liposomes compared with RC. From this we conclude that RC is a better choice for optimization. As shown in Table S13, the two most common MWCO values were compared, 10 kDa and 30 kDa. The principal effect of MWCO is on retention time and channel pressure during elution (data not show). Otherwise, the results and separation efficiencies appear similar. Based on this data, we conclude that either MWCO value will work for separation of liposomal drugs; 10 kDa was chosen for further optimization because it is more commonly employed for separations involving nanomaterials.

#### 3.2.4 Mobile phase

Mobile phase composition is likely the most critical variable with respect to optimizing an AF4 method. It impacts nearly every aspect of the fractionation process, and the analyst has a wide range of options to choose from in order to enhance method performance while minimizing alteration to the analyte during the fractionation process. Unless otherwise stated, the following conditions were applied during evaluation of the mobile phase: short channel, 350 μm spacer, 10 kDa RC membrane, DF = 0.5, FF = 2, XF = 0.3 and 25 μg injected mass. For NEPs, it is important to measure their properties in a representative state, so an isotonic mobile phase at physiological pH is generally recommended. In the present study, fractionation was evaluated using unbuffered isotonic saline (pH 6) and PBS buffers (pH 7.2 to 7.4) purchased from different sources and which are characterized by slightly different chemical compositions (see Table S1 for details). The results, reported in Fig. 6D and in Table S14, show that all tested mobile phase compositions yield similar or nearly identical results, based on sample recovery, separation efficiency and the measured sizes. PBS was selected here as the principal mobile phase because it provides near isotonic dilution of the liposomal formulations (avoiding potential adverse osmotic effects, such as impacting release of the active pharmaceutical ingredient), its pH is in the physiologic range and it is chemically stable. Additionally, PBS has been widely used as a dilution medium for studies of liposomal drug formulations reported in the literature.

*Critical Point – While the composition of PBS media can vary, and pH can range from 7.2 to 7.5, PEGylated liposomes exhibit nearly identical fractionation behavior regardless of these variations. We conclude that the source of PBS is not a significant factor, at least within the range evaluated here.*

#### 3.2.5 Focus and relaxation

The focus time and FF are critical parameters for an MD-AF4 method. If focusing is not appropriately set, the separation can be directly affected, and results misinterpreted. The processes involved during injection/focus/relaxation are crucial for the subsequent elution/fractionation/detection processes. A poorly optimized focus/relaxation step can result in sample loss, a void peak with substantial entrainment of analyte and distorted or broadened peaks. The focus/relaxation step was optimized by analyzing FF and the focusing time. Unless otherwise stated, the following conditions were applied during optimization of focus and relaxation: PBS mobile phase, short channel, 350 μm spacer, 10 kDa RC membrane, DF = 0.5, XF = 0.3 and 25 μg injected mass.

Initially, FF was varied from 1 to 2 with no observable effect (data not shown). Then FF was fixed at 2 and focus times of (3, 5, and 8) min were tested. Surprisingly, results (Fig. 6E and Table S15-S16) show that focus time has virtually no impact on fractionation of Dox1, so a focus time of 8 min was selected for the optimized method to ensure the formation of a uniform analyte band prior to elution. However, results indicate that shorter focus times can be applied in situations where 8 min is observed to induce agglomeration in the channel and/or result in low recovery. We emphasize that these effects were not observed for the PEGylated liposomes investigated in the present study.

#### 3.2.6 Sample mass

The mass of injected analyte (based on lipid content) impacts the fractionation process as well as the detector response. If the mass is too low, detector sensitivity will be compromised. If the mass is too high (referred to as overloading), detectors will saturate and fractionation can be adversely affected (e.g., coelution, where different size particles elute together instead of at different retention times or retained material that is not fractionated). The correct range will depend to some extent on the optical scattering properties and size of the analyte and is best evaluated by injecting a series of known masses under conditions appropriate for the intended analysis. The resulting fractograms can then be compared with respect to detector signal/noise and peak quality as well as analyte recovery. The objective is to identify a mass range that provides sufficient signal/noise, without saturating detectors or inducing memory effects and/or compromised recovery. Unless otherwise stated, the following conditions were applied during optimization of injected mass: PBS mobile phase, short channel, 350 μm spacer, 10 kDa RC membrane, DF = 0.5, FF = 2 and XF = 0.3.

**Fig. 7:**
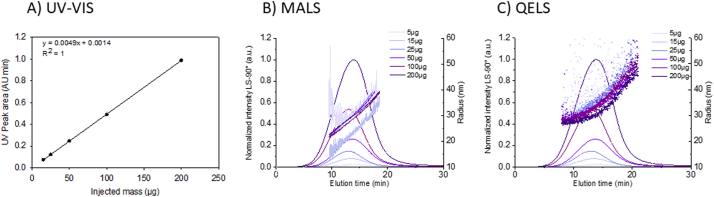
Injected mass series. Test of the effect of Dox1 injected mass from (5 to 200) μg. A) UV-VIS linear response at 280 nm reporting the area of the elution peak as a function of the injected mass B) Fractograms (normalized excess Rayleigh ratio at 90°), and calculated R_g_ (line) from the MALS detector and C) R_h_ (dots) measured by the QELS detector. The shift from light to dark purple represents the increase of injected mass. (For interpretation of the references to colour in this figure legend, the reader is referred to the web version of this article.)

As shown in Fig. 7(B—C), for injected mass of Dox1 < 15 μg, the noise of the scattering intensity trace registered by either MALS or QELS sensors is high, even across the peak FWHM, thereby degrading the capacity to measure reliable size values (Table S17). Alternatively, no loss of resolution or reduction in t_R_ due to channel overloading was observed for the conditions tested at injected mass up to 200 μg. It is notable that *R*_h_ degrades (becomes noisier) faster with decreasing mass relative to MALS derived *R*_g_, an effect observed for other analytes as well. On the other hand, the response of the mass sensitive UV–Vis signal at 280 nm (Fig. 7A) is linear over the tested range. To minimize sample consumption, avoid potential memory effects and maintain an appropriate signal/noise for light scattering detectors, (25 to 50) μg was identified as the optimal range for injected mass of liposomal formulations.

*Critical Point – Although injected mass must be controlled, the injected volume can vary since excess liquid will be removed through the membrane during injection/focusing. Therefore, if sensitivity is an issue for a low concentration sample, a larger volume can be injected to obtain more analyte mass.*

### 3.3 MD-AF4 optimized method summary

The liposome formulation should first be diluted in the mobile phase to a lipid concentration of 1 mg/mL and stored refrigerated until needed for analysis; the dilution factor will depend on the native concentration, but for the liposomes used in this study it ranged from 15× to 20×. The optimized fractionation method derived from the previously described results consists of an 8 min focus plus injection step at FF = 2.0, followed by elution with a constant XF = 0.3 applied for 45 min with a DF = 0.5 and a final elution without XF for 15 min, as summarized in Table 1. The trapezoidal long channel (Wyatt or Postnova) is equipped with a 10 kDa RC membrane and a spacer of 350 μm with wide width. A lipid mass of 25 μg is injected. To minimize potential memory or contamination effects, a washing step between each liposome analysis is conducted. In the washing procedure, 50 μL of PBS mobile phase is injected with an 8 min focus step at a flow rate of 2.0, followed by a 13 min elution without XF at DF = 0.5, as described in SI Section 4. The injection volume for washing should meet or exceed the injection volume used for samples.

**Table 1:** Optimized AF4 fractionation method for liposomal formulations.

Channel parameters	Channel length	Long channel (280 mm)	
	Spacer	350 μm with wide width	
Membrane	Type	Regenerated cellulose (RC)	
	MWCO	10 kDa	
Flow rates	Injection flow	0.2 mL/min	
	Channel flow	0.5 mL/min	
	Focus flow	2 mL/min	
	Cross flow	0.3 mL/min	
Sample loading	Injection amount	25 μg	
Time and flow parameters (as sequenced in the method)	Mode	Step duration (min)	XF (mL/min)
	(1) Elution	2	0
	(2) Focus	2	–
	(3) Focus + Injection	8	–
	(4) Elution	45	0.3
	(5) Elution	15	0

### 3.4 Method precision, reproducibility and variability

**Fig. 8:**
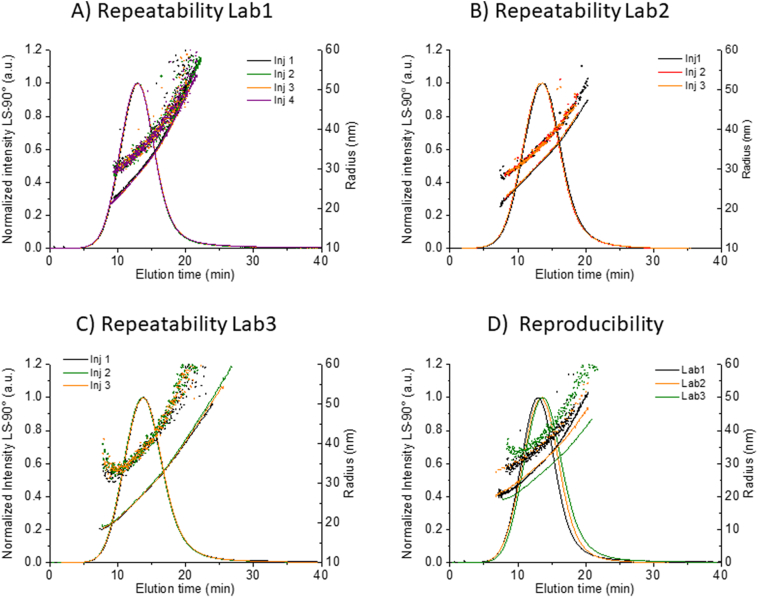
Repeatability and reproducibility of the optimized method for Dox1. Fractograms (normalized excess Rayleigh ratio at 90°), Rg (line) and Rh (dots) for replicate Dox1 injections using the same method on different platforms and labs. Repeatability of A) four replicate injections performed by Lab1 (Wyatt Eclipse Dualtec), B) four replicate injections performed by Lab2 (Wyatt Eclipse 4), C) three replicate injections performed by Lab3 (Postnova AF2000) and D) reproducibility of the optimized method, comparing a single injection by Lab1 (black), Lab2 (orange) and Lab3 (green). (For interpretation of the references to colour in this figure legend, the reader is referred to the web version of this article.)

Method precision was evaluated under repeatability conditions (same analyst, same instrument, same location, same day), by analyzing at least 3 replicate injections of Dox1 in each of three laboratories (see SI Section 1 for detailed description of instrumentation used in each laboratory). The calculated means and standard deviations are summarized in Table 2, while representative fractograms are shown in Fig. 8 (Fig. S6). The coefficient of variation (COV) of the repeatability inside each lab does not exceed 5% for any relevant parameter including recovery, *t*_R_ and the calculated size, demonstrating excellent repeatability for the method on all platforms involved in this study. The COV for *R* is relatively high, due principally to variations in t_0_ and the very low ratios measured in this study (0.02 < *R* < 0.04). A low ratio is indicative of a highly efficient fractionation, where the optimal range is approximately 0.03 ≤ *R* ≤ 0.2 [43]. Due to the significant effect of the variation of the void time on *R*, the value of *R* was not considered a key parameter for the evaluation of method precision or reproducibility, but the low values are indicating overall of efficient fractionation.

**Table 2:** Repeatability and reproducibility of the optimized method applied to Dox1. R_h_ measured by QELS (Wyatt) at scattering angles of 99.9° (Lab1) or 134° (Lab2) or by Zetasizer (Malvern) in flow mode at 173° (Lab3). The mean COV of at least 3 replicate injections is reported for each parameter. For size, the FWHM mean and spread is reported.

Platform	Replicates	*R*% (%)	*t*_R_ (min)	*R*_g_ (nm)	*R*_g_ Spread (nm)	*R*_h_ (nm)	*R*_h_ Spread (nm)	*R*_g_/*R*_h_
Lab1	5	98 (1)	11.9 (2.5%)	28.9 (1%)	11.9 (5%)	34.2 (1%)	11.0 (5%)	0.84 (2%)
Lab2	3	95 (1)	13.1 (3.8%)	30.2 (1%)	11.8 (2%)	35.9 (0.5%)	11.3 (4%)	0.83 (4%)
Lab3	3	96 (1)	12.7 (1%)	27.6 (1%)	11.7 (4%)	38.9 (0.5%)	11.7 (5%)	0.71 (3%)
All mean	11	96	12.6	28.9	11.8	36.3	11.3	0.79
All COV		2%	4.8%	4.5%	1%	6.5%	3%	9%

Reproducibility was assessed by applying the method for Dox1, from sample preparation through measurement and data analysis, at three independent facilities using three different AF4 instrument platforms (see SI Section 1 for details) and analysts. The mean and COV values for all endpoints are summarized in Table 2, and demonstrate the excellent repeatability obtained within labs and the very good reproducibility obtained between labs and platforms. For most measurands, such as *R%*, *t*_R_ and *R*_g_ the COV is less than 5%. The COVs for reproducibility of *R*_h_ and of *R*_g_/*R*_h_ are estimated to be 6.5% and 9%, respectively, suggesting that the different online DLS instrumental configurations (e.g., angle, acquisition time, online cell volume) and the different data analysis approaches (single exponential vs. cumulants analysis of the measured correlation function) are significant sources of bias for online determination of *R*_h_.

**Fig. 9:**
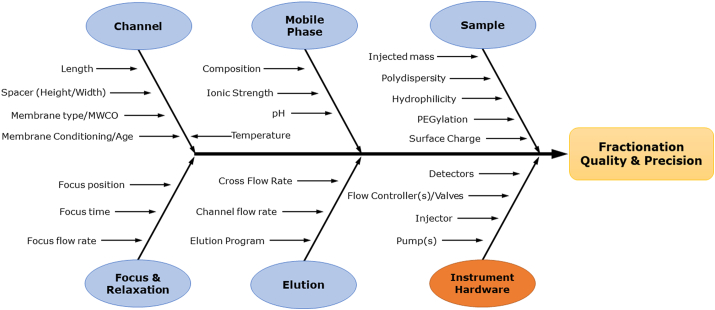
Cause and effect diagram showing the principal components of fractionation quality and variability for the analysis of liposomal drug formulations*.* Membrane age refers to effects that appear over time and repeated use of the same membrane, whereas conditioning refers to pretreatment with sample or other complex media prior to analyte injection.

Finally, the principal factors impacting variability in fractionation of liposomal drug formulations are summarized schematically in a cause and effect diagram (Fig. 9). These factors can be grouped into six components of the measurement process, namely the channel, mobile phase, sample, focus/relaxation, elution and instrument hardware. Of these, only the first five are considered adjustable or selectable by the analyst. The last one, hardware, is dependent on the correct operation of the instrument components according to the manufacturer's specifications. Variations, fluctuations, errors or operational misfunction of any of these factors can contribute to poor quality and/or variability in the fractionation process and results. Tracking the cause of variability or poor performance is often a matter of trial and error, though following the guidelines set out in ISO TS 21362, Gigault et al. 2014 [43] and the optimization process described above can accelerate the process.

### 3.5 Method validation

#### 3.5.1 Polydisperse liposomal formulation

**Fig. 10:**
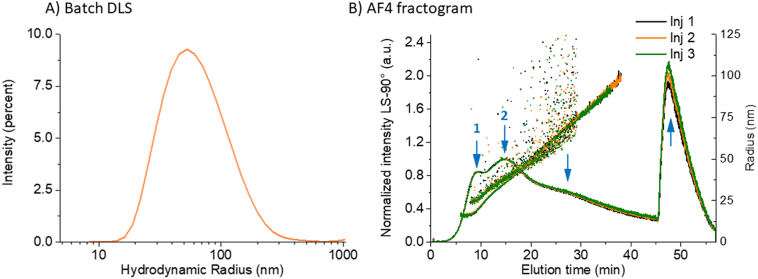
Dox2: Batch DLS vs AF4 analysis using the optimized method. A) Batch DLS of Dox 2, B) Fractograms showing the elution profile of three replicate injections (normalized excess Rayleigh ratio at 90°), R_g_ (line) and R_h_ (dots)versus elution time after injecting 25 μg of Dox2. Blue arrows indicate identifiable component populations. (For interpretation of the references to colour in this figure legend, the reader is referred to the web version of this article.)

This phase of validation challenged the method using a polydisperse/multimodal research-grade doxorubicin HCl-liposomal formulation (Dox2), one that presents a substantially more complex sample with physical characteristics substantially different from the reference listed drug, but with a composition that is nominally the same. Screening with batch mode DLS indicated a PI of 0.26 (Fig. 10 and Table S4) compared with <0.05 for Dox1. The MD-AF4 analysis of Dox2 (Fig. 10) shows that the sample contains at least three populations (and possibly four) that were not resolved by batch mode DLS. The first two eluted peaks are associated with spherical vesicles (Peak 1 mode *R*_g_ = 20 nm, Peak 2 mode *R*_g_ = 36 nm). A prominent third population eluting after removal of the XF (i.e., the “retained peak” eluting after 45 min), is characterized by a mixture of coeluting, very large entities, and based on initial cryo-TEM results (data not shown), appears to contain predominantly elongated narrow dark objects that resemble free crystals of doxorubicin HCl. Further work would be necessary to confirm this. One could extend the elution run in this case to fully elute this retained material if desired. Additionally, there is a broad shoulder centered near 30 min, that could represent a forth population, though poorly defined at best.

Overall, the optimized method offers flexibility to capture the polydispersity, multimodal state and other subtle but possibly important characteristics of this complex liposomal sample. To evaluate the sensitivity of the sample response (e.g., resolution, peak quality, detector signal, recovery) to variations in key method parameters, we examined the effects of elution program, channel spacer, focusing time and injected mass. These results are shown graphically in Fig. S4 and summarized below. The effect of elution (XF) programming and spacer thickness had the greatest impact on the polydisperse Dox2. Specifically, as with the monomodal liposomes, a constant XF is most efficient. The overall fractionation quality changes much more drastically (relative to Dox1) with a change in spacer thickness (350 μm shows the best results). Reducing the focusing time or FF did not improve mass recovery for Dox2, nor did it reduce the size of the retained peak (Fig. S4). This reinforces the conclusion that the retained peak is not an artefact induced during the focusing and relaxation phase, but a population of objects characterized by different physical properties and highlight the capability of the method to answer various liposome related questions. Most notably, the effect of injected mass on the MALS and QELS response (Fig. S5 and Table S18) data suggests that this parameter might need to be modified for polydisperse samples in order to improve the data quality (signal).

*Critical Point – For a monomodal sample the analyte mass is concentrated in a relatively narrow band, whereas with a polydisperse sample the mass is widely dispersed as a result of which the detector sensitivity is reduced locally. The injected mass of polydisperse samples can be adjusted, if necessary, to obtain an acceptable signal for data analysis across all components.*

Examination of injected mass over the range from (15 to 200) μg showed a pronounced memory effect for Dox2 in the channel (despite the use of a washing step between injections (SI Section 4)). An injected mass of 25 μg produced the best compromise between signal quality and memory effect.

Overall it was demonstrated that the MD-AF4 method optimized for Dox1 was also appropriate to analyze more complex liposomal samples, such as Dox2. Again, repeatability under the optimized method was excellent (Tables S18 and S19, Fig. S6).

#### 3.5.2 Generic Doxil formulations

**Fig. 11:**
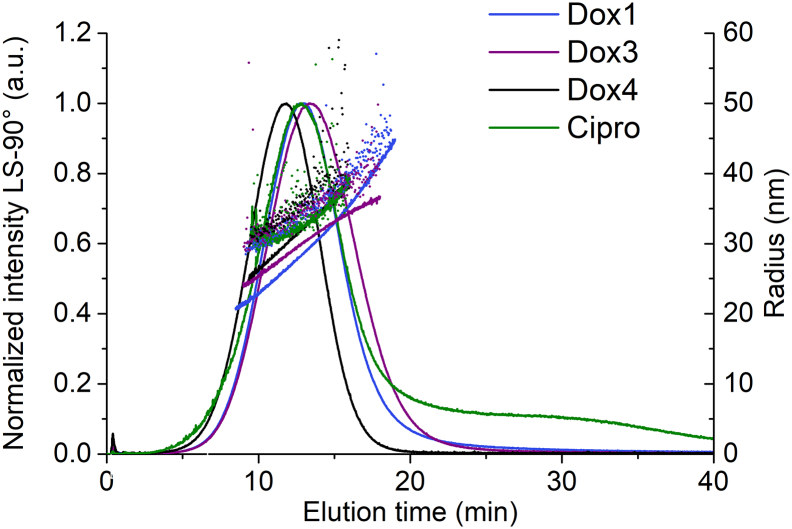
Application of the optimized method to liposomal formulations. Comparison of the representative fractograms (normalized excess Rayleigh ratio at 90°), R_g_ (line) and R_h_ (dots) obtained for Dox1 (violet), Dox3 (purple), and Dox4 (black). and Cipro (green). (For interpretation of the references to colour in this figure legend, the reader is referred to the web version of this article.)

The method has been demonstrated to be precise and reproducible for the prototype doxorubicin HCl-PEG/liposomal formulation Dox1 (identical to the FDA reference listed drug, Doxil®) and its drug-free control Dox1C (see e.g., Fig. S7). To further validate the method, applicability to two FDA approved generics of the reference listed drug (identified as Dox3 and Dox4) was assessed. Representative fractograms for Dox3 and Dox4 are shown and compared with Dox1 in Fig. 11. The method performs well with excellent recoveries (> 90%) and repeatability for the two FDA approved generics. Moreover, the approach yields results for the generics that are consistently similar to Dox1, with very small shifts in retention time (see Table S19). Like Dox1, Dox3 and Dox4 are characterized by a single population of spherical vesicles eluting at retention time 12 min with a very small void peak indicating no significant unretained analyte. Dox1 and the generics yield mean *R*_g_ (Berry model) and *R*_h_ (QELS, 99.9°) values near 30 nm and 35 nm, respectively. The size spread for all three Dox samples is roughly 10 nm and the shape factor falls between 0.84 and 0.86. As with Dox1, for the generics the COV does not exceed 5% for any relevant parameter, demonstrating excellent within-lab precision for multiple related liposomal products. The repeatability under the optimized method for the two generic Doxil formulations was excellent (Fig. S6).

#### 3.5.3 Liposomal ciprofloxacin

Further validation of the method was demonstrated by application to a research-grade PEGylated liposomal antibiotic drug identified here as Cipro (PEGylated hydrogenated soybean phosphatidylcholine liposomal ciprofloxacin). Like Dox1, Cipro is also characterized by a monomodal population with a mean *R*_g_ at 32 nm (Fig. 11). The shape factor for Cipro (Table S19) is slightly higher approaching the theoretical value (1) for hollow spheres (0.93 vs. 0.84–0.88 for Dox1, Dox2 and Dox3), suggesting that the Cipro liposomes might possess a less dense core than Dox liposomes. Cipro also shows a lingering tail at longer elution times. Beyond these details, the results again demonstrate that the optimized method is widely applicable for PEGylated liposomal formulations in the nanosize range. Large liposomes (2*R*_h_ > 200 nm) will be retained and will elute as a distorted peak following the removal of XF. These larger particles also produce unreliable DLS results online, even at relatively low DF. The repeatability under the optimized method was excellent (Fig. S6).

#### 3.5.4 Application in complex media

As recently reported by the FDA [46], when NEPs are evaluated, in addition to characterizing the physico-chemical properties of the analyte, in order to fully assess their quality, safety and efficacy profile it is also necessary to measure their stability and interactions in complex protein-containing biological media. In fact, when NEPs are entering the systemic circulation system, the interaction of plasma proteins with their surface may endow NEP systems with new properties, e.g. modifying their surface (formation of protein corona), size (formation of corona, agglomeration, dissolution) and drug delivery (impact drug release). The investigation of size stability in complex biological media is a difficult challenge that requires the use of high-resolution techniques [12]. MD-AF4 is a very powerful method to measure NEP-protein interactions, thanks to the separation of free proteins from the analyte particles prior to analysis. This has previously been demonstrated for liposomal samples [22] and lipid-based nanoparticles [28].

Herein the capacity of the optimized MD-AF4 method to analyze the size of Dox1 and Dox2 in the presence of serum proteins was evaluated. For this purpose, the liposomal formulations were diluted into 10% buffered fetal bovine serum (FBS), and immediately analyzed using the optimized method without further incubation.

**Fig. 12:**
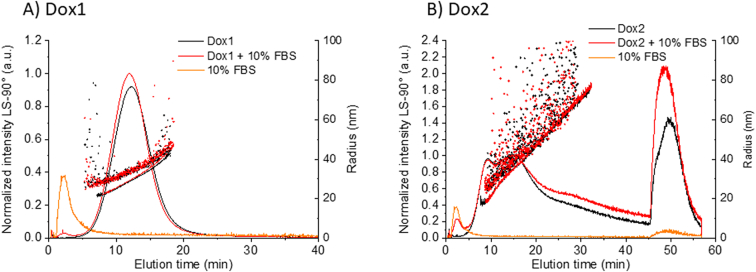
Measurement of Dox1 and Dox2 in complex media. AF4 fractograms (normalized excess Rayleigh ratio at 90°), the R_g_ (line) and R_h_ (dots) versus elution time by injecting A) Dox1 and B) Dox2 with (red) and without (black) 10% FBS. Orange fractogram is the PBS + 10% FBS control. (For interpretation of the references to colour in this figure legend, the reader is referred to the web version of this article.)

The results for the fractionation of liposomal doxorubicin samples Dox1 and Dox2 in simple and complex media are reported in Fig. 12 and Table S20. The optimized elution program successfully separates free protein (see control fractogram of buffered FBS in yellow) from the liposome component. Under the conditions tested, the fractograms of the liposomes observed following dilution into 10% FBS are phenomenologically similar to the liposomes without protein-containing FBS (i.e., the same principle features appear at roughly the same elution times). Overall, we can conclude that the optimized MD-AF4 method has the potential to analyze liposomal samples in complex biological media as well as simple PBS.

*Critical point: This experiment provides proof of principle, while actual preclinical studies of liposomal drug interactions in complex media should include appropriate incubation periods prior to analysis.*

### 3.6 Method limitations and troubleshooting

The method described here should work well with any PEGylated liposomal drug formulation as well as their controls (empty liposomes). Liposomes that do not contain PEG in their outer shell may exhibit stability issues and/or substantial membrane interactions in the AF4 channel. These materials should be evaluated on a case-by-case basis. But generally, any liposome that has a hydrophilic, negatively charged or neutral, external surface and is stable in PBS or saline, should be applicable.

As stated previously, online DLS measurements have limitations with respect to the upper size range, especially at relatively high DF values. This limitation is most evident in the standard MALS cell and less so in the type of cell used with a Zetasizer in flow-mode operation. A larger bore MALS cell can mitigate this limitation for particles larger than about 75 nm (radius). The root mean square radius is limited on the low end to about 10 nm, but this limit is a function of the laser wavelength and angular range available on a specific system. The upper limit for *R*_g_ is well beyond the size range of interest for this method. We strongly suggest the analyst confirm the applicable size range for each specific online detector configuration by analyzing size standards or quality control materials, and to avoid reporting size values outside of this range.

Highly polydisperse or multimodal samples present the greatest challenge for application of this method. But as demonstrated with Dox2, the method is sufficiently flexible to capture basic information regarding the physical state of complex analytes. Simple adjustments, such as using a longer elution time, can be made within the scope of this method in order to ensure that all components are fractionated and analyzed. Samples containing very large liposomes or liposome products (e.g., agglomerates) greater than about 200 nm radius, are outside the scope of this method and its intended application to drug formulations.

Low mass recovery (less than 90% for PEGylated liposomes) indicates that the analyte is interacting strongly with the membrane (accumulation wall), though losses can also occur at other surfaces in contact with the fluid sample. Reducing FF, focus time and XF during elution can improve recovery. However, very low recoveries (< 70%) that cannot be improved by the usual approach most likely disqualify the sample as incompatible with the method, and further suggest that the material properties are inappropriate for clinical applications. An unexpected low recovery for a formulation that has proven highly recoverable in the past is a red flag suggesting a change has occurred as a result of, for example, aging, storage, or a manufacturing quality control.

*Critical point – An alternative approach to improve recovery for high-loss analytes is to passivate the membrane with a suitable coating such as a surfactant or protein. Use of passivation must be validated as part of the overall method performance.*

Low or noisy detector response can usually be addressed by increasing the injected analyte mass. Low or noisy signals might occur, for instance, in a polydisperse sample where the mass is spread out over a larger elution time. If increasing the injection mass does not eliminate the problem, the detector itself should be evaluated or the cell cleaned and retested. On the other hand, a high MALS baseline signal suggests the cell is fouled and requires cleaning. Comparison with a known quality control material such as PSL is recommended.

Memory effects occur when components from a previously injected sample elute during a new injection. The most likely cause is material adhering to the membrane or other surfaces, then releasing during the second elution. If the memory effect is substantial (i.e., it significantly interferes with the outcome of an analysis), there are steps that can be taken to eliminate or minimize the effect. In the present work, the method includes a mobile phase injection (without cross flow) before each sample injection. This approach can be extended if necessary. For instance, multiple mobile phase injections could be performed prior to each sample injection. Memory effects are usually associated with recovery issues, so improving recovery can help eliminate memory effects.

*Critical point – The mass detector or scattered intensity signal should be monitored during the washing step, and will indicate whether or not additional washing steps are necessary between samples*

*Best Practice Note – The lifetime of a membrane depends on the number of injections, the time over which analyses are performed, the duration of the washing/elution steps, and the quantity of mass injected per analysis. Taking all factors into consideration, a membrane can typically be used for 30–50 analyses of liposomal samples before replacement is necessary. This number can vary widely for different types of samples and experimental conditions. If memory effects persist after several washing cycles, then the membrane should be replaced.*

The analyst should always monitor channel pressure at all stages of the experiment and look for any unexpected changes in pressure (typically increases). The system should automatically terminate an experiment if the pressure exceeds the manufacturer's stated operational range. But any sudden or unexpected increase in pressure should be investigated immediately. The cause can range from a clogged membrane or fluid line to a bad valve or flow controller.

## 4 Summary and Conclusions

**Fig. 13:**
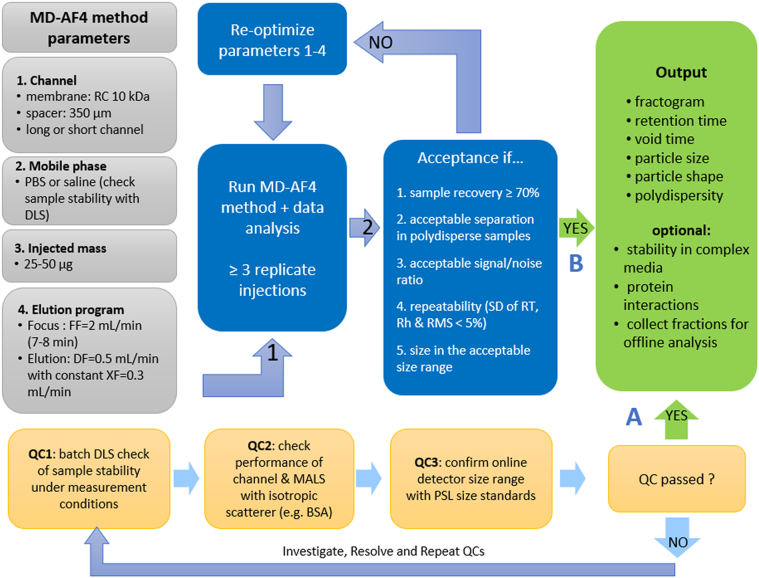
Work flow chart showing principal steps in MD-AF4 method application. Both quality control (A) and fractionation/analysis results (B) must yield green arrows to meet overall method acceptance criteria, with QC preceding sample analysis.

In this extensive multi-laboratory investigation, we evaluated a broad range of instrument parameters and experimental factors that influence the outcome of multi-detector asymmetrical-flow field flow fractionation (MD-AF4) applied to the physical characterization of nanometric liposomal drug formulations. Liposomal doxorubicin HCl was utilized as the prototypical test material for this study. The principal outcome of this work is an optimized MD-AF4-based method (schematically illustrated in Fig. 13) validated across different instrument platforms, three laboratories, multiple drug formulations and in complex media containing serum proteins. The method was evaluated for a research grade form of the reference listed drug Doxil, a research grade polydisperse formulation, two pharmaceutical generics of Doxil and a research grade ciprofloxacin liposome. All test materials were PEGylated.

Key parameters and factors were identified and optimized in order to yield reliable and accurate results with respect to fractionation quality, particle size and size distribution, particle shape, recovery, separation efficiency and selectivity. Interlaboratory reproducibility and intralaboratory precision were evaluated. The resulting method proved efficient and sufficiently flexible to tackle a range of physical states and formulations. We observed that small variations in phosphate buffered saline composition (common in commercial products) did not significantly impact results, and that formulations can be analyzed in complex media without detriment to the outcome. In fact, results show that this method has the potential to provide additional information regarding interactions between the drug and medium components such as serum proteins. Additionally, the potential for quantifying and discriminating between encapsulated and free drug using MD-AF4, coincidentally with analysis of the physical state of the drug, is proposed and under further investigation.

This method meets a documented high priority need in regulatory science as applied to nano-enabled medical products, and has substantial advantages over commonly used batch dynamic light scattering (DLS), particularly in the case of polydisperse and multimodal complex formulations, where DLS results can be both misleading and difficult to interpret. In addition to liposomal formulations, we believe the method can be easily adapted to the measurement of other NEPs (such as lipid nanoparticles).

Overall, this optimized method will help accelerate development of NEPS, and facilitate their regulatory review, ultimately leading to faster translation of innovative concepts from the bench to the clinic. This method will form the basis for a future international consensus test method, that will involve a more extensive interlaboratory comparison. Additionally, the approach used in this work (based on international collaboration between leading non-regulatory institutions) can be replicated to address other identified gaps in the analytical characterization of various classes of NEPs.

## Appendix A Supplementary data

Supplementary material Image 1

## Footnotes

1 The identification of any commercial product or trade name does not imply endorsement or recommendation by the National Institute of Standards and Technology

2 Typically, channel and detector flow are identical, but, in some configurations, it is possible for detector flow to differ from channel flow, for instance when a post-channel or within channel splitter is used. In this study they are interchangeable terms.

## References

[1] Anselmo A.C., Mitragotri S. (2016). Nanoparticles in the clinic. Bioeng. Transl. Med..

[2] D'Mello S.R., Cruz C.N., Chen M.-L., Kapoor M., Lee S.L., Tyner K.M. (2017). The evolving landscape of drug products containing nanomaterials in the United States. Nat. Nanotechnol..

[3] Farokhzad O.C., Langer R. (2009). Impact of nanotechnology on drug delivery. ACS Nano.

[4] Myshko D. (2004). Nanotechnology: It’s a Small World.

[5] Park K. (2019). The beginning of the end of the nanomedicine hype.

[6] Grodzinski P. (2019). NCI Centers of Cancer nanotechnology excellence (CCNEs) - a full story to set the record straight. J. Control. Release.

[7] Cryer A.M., Thorley A.J. (2019). Nanotechnology in the diagnosis and treatment of lung cancer. Pharmacol. Ther..

[8] Anchordoquy T.J., Barenholz Y., Boraschi D., Chorny M., Decuzzi P., Dobrovolskaia M.A., Farhangrazi Z.S., Farrell D., Gabizon A., Ghandehari H. (2017). Mechanisms and Barriers in Cancer Nanomedicine: Addressing Challenges, Looking for Solutions.

[9] Grossman J.H., Crist R.M., Clogston J.D. (2017). Early development challenges for drug products containing Nanomaterials. AAPS J..

[10] Swierczewska M., Crist R.M., McNeil S.E. (2018). Evaluating Nanomedicines: Obstacles and advancements. Characterization of Nanoparticles Intended for Drug Delivery.

[11] Tyner K.M., Zou P., Yang X., Zhang H., Cruz C.N., Lee S.L. (2015). Product quality for nanomaterials: current US experience and perspective. Wiley Interdiscip. Rev: Nanomed. Nanobiotechnol..

[12] Gioria S., Caputo F., Urbán P., Maguire C.M., Bremer-Hoffmann S., Prina-Mello A., Calzolai L., Mehn D. (2018). Are existing standard methods suitable for the evaluation of nanomedicines: some case studies. Nanomedicine.

[13] Halamoda-Kenzaoui B., Baconnier S., Bastogne T., Bazile D., Boisseau P., Borchard G., Borgos S.E., Calzolai L., Cederbrant K., Di Felice G. (2019). Bridging communities in the field of nanomedicine. Regul. Toxicol. Pharmacol..

[14] Bremer-Hoffmann S., Halamoda-Kenzaoui B., Borgos S.E. (2018). Identification of regulatory needs for nanomedicines. J. Interdiscip. Nanomedicine.

[15] Halamoda-Kenzaoui B., Holzwarth U., Roebben G., Bogni A., Bremer-Hoffmann S. (2019). Mapping of the available standards against the regulatory needs for nanomedicines. Wiley Interdiscip. Rev.: Nanomed. Nanobiotechnol..

[16] Global Summit on Regulatory Science (GSRS16) (2016). Nanotechnology Standards and Applications - Final Report.

[17] Food U., Administration D. (2018). Liposome drug products: Chemistry, manufacturing, and controls; human pharmacokinetics and bioavailability; and Labeling documentation. Guidance for industry. Guidance for Industry.

[18] Calzolai L., Gilliland D., Garcìa C.P., Rossi F. (2011). Separation and characterization of gold nanoparticle mixtures by flow-field-flow fractionation. J. Chromatogr. A.

[19] Mehn D., Caputo F., Rösslein M., Calzolai L., Saint-Antonin F., Courant T., Wick P., Gilliland D. (2017). Larger or more? Nanoparticle characterisation methods for recognition of dimers. RSC Adv..

[20] Caputo F., Arnould A., Bacia M., Ling W.L., Rustique E., Texier I., Mello A.P., Couffin A.-C. (2019). Measuring particle size distribution by asymmetric flow field flow fractionation: a powerful method for the preclinical characterization of lipid-based nanoparticles. Mol. Pharm..

[21] Caputo F., Clogston J., Calzolai L., Rösslein M., Prina-Mello A. (2019). Measuring particle size distribution of nanoparticle enabled medicinal products, the joint view of EUNCL and NCI-NCL. A step by step approach combining orthogonal measurements with increasing complexity. J. Control. Release.

[22] Mehn D., Caputo F., Rösslein M. (2017). EUNCLPCC-022. - measurement of particle size distribution of protein binding, of mean molecular weight of polymeric NP components. Study of Batch to Batch Reproducibility, and Study of Release of Free Coating from NP Surface by FFF-MALS.

[23] Marioli M., Kok W.T. (2019). Recovery, overloading, and protein interactions in asymmetrical flow field-flow fractionation. Anal. Bioanal. Chem..

[24] Wagner M., Holzschuh S., Traeger A., Fahr A., Schubert U.S. (2014). Asymmetric flow field-flow fractionation in the field of nanomedicine. Anal. Chem..

[25] Contado C. (2017). Field flow fractionation techniques to explore the “nano-world”. Anal. Bioanal. Chem..

[26] Kuntsche J., Decker C., Fahr A. (2012). Analysis of liposomes using asymmetrical flow field-flow fractionation: separation conditions and drug/lipid recovery. J. Sep. Sci..

[27] Iavicoli P., Urbán P., Bella A., Ryadnov M.G., Rossi F., Calzolai L. (2015). Application of asymmetric flow field-flow fractionation hyphenations for liposome–antimicrobial peptide interaction. J. Chromatogr. A.

[28] Hupfeld S., Ausbacher D., Brandl M. (2009). Asymmetric flow field-flow fractionation of liposomes: 2. Concentration detection and adsorptive loss phenomena. J. Sep. Sci..

[29] Hinna A., Steiniger F., Hupfeld S., Brandl M., Kuntsche J. (2014). Asymmetrical flow field-flow fractionation with on-line detection for drug transfer studies: a feasibility study. Anal. Bioanal. Chem..

[30] Van Haute D., Jiang W., Mudalige T. (2019). Evaluation of size-based distribution of drug and excipient in amphotericin B liposomal formulation. Int. J. Pharm..

[31] Hupfeld S., Moen H.H., Ausbacher D., Haas H., Brandl M. (2010). Liposome fractionation and size analysis by asymmetrical flow field-flow fractionation/multi-angle light scattering: influence of ionic strength and osmotic pressure of the carrier liquid. Chem. Phys. Lipids.

[32] Monteiro L.O., Malachias A.n., Pound-Lana G., Magalhães-Paniago R.r., Mosqueira V.C., Oliveira M.n.C., de Barros A.L.s.B., Leite E.A. (2018). Paclitaxel-loaded pH-sensitive liposome: new insights on structural and physicochemical characterization. Langmuir.

[33] Evjen T.J., Hupfeld S., Barnert S., Fossheim S., Schubert R., Brandl M. (2013). Physicochemical characterization of liposomes after ultrasound exposure – mechanisms of drug release. J. Pharm. Biomed. Anal..

[34] Zhang H., Freitas D., Kim H.S., Fabijanic K., Li Z., Chen H., Mark M.T., Molina H., Martin A.B., Bojmar L., Fang J., Rampersaud S., Hoshino A., Matei I., Kenific C.M., Nakajima M., Mutvei A.P., Sansone P., Buehring W., Wang H., Jimenez J.P., Cohen-Gould L., Paknejad N., Brendel M., Manova-Todorova K., Magalhães A., Ferreira J.A., Osório H., Silva A.M., Massey A., Cubillos-Ruiz J.R., Galletti G., Giannakakou P., Cuervo A.M., Blenis J., Schwartz R., Brady M.S., Peinado H., Bromberg J., Matsui H., Reis C.A., Lyden D. (2018). Identification of distinct nanoparticles and subsets of extracellular vesicles by asymmetric flow field-flow fractionation. Nat. Cell Biol..

[35] Zhang H., Lyden D. (2018). A protocol for asymmetric-flow field-flow fractionation (AF4) of small extracellular vesicles. Protocol Exch..

[36] Barenholz Y.C. (2012). Doxil®—the first FDA-approved nano-drug: lessons learned. J. Control. Release.

[37] Bavli Y., Winkler I., Chen B.M., Roffler S., Cohen R., Szebeni J., Barenholz Y. (2019). Doxebo (doxorubicin-free Doxil-like liposomes) is safe to use as a pre-treatment to prevent infusion reactions to PEGylated nanodrugs. J. Control. Release.

[38] Barenholz Y.C. (23 October 2019). Hebrew University Hadassah Medical School, in, Personal Communication.

[39] Andersson M., Wittgren B., Wahlund K.-G. (2003). Accuracy in multiangle light scattering measurements for molar mass and radius estimations. Model calculations and experiments. Anal. Chem..

[40] Wyatt P.J. (1993). Light scattering and the absolute characterization of macromolecules. Anal. Chim. Acta.

[41] Burchard W., Schmidt M., Stockmayer W.H. (1980). Information on Polydispersity and branching from combined quasi-elastic and Intergrated scattering. Macromolecules.

[42] ISO/TS 21362:2018 (2018). Nanotechnologies — Analysis of nano-objects using asymmetrical-flow and centrifugal field-flow fractionation.

[43] Gigault J., Pettibone J.M., Schmitt C., Hackley V.A. (2014). Rational strategy for characterization of nanoscale particles by asymmetric-flow field flow fractionation: a tutorial. Anal. Chim. Acta.

[44] Sitar S., Vezočnik V., Maček P., Kogej K., Pahovnik D., Žagar E. (2017). Pitfalls in size characterization of soft particles by dynamic light scattering online coupled to asymmetrical flow field-flow fractionation. Anal. Chem..

[45] Litzen A., Wahlund K.G. (1991). Zone broadening and dilution in rectangular and trapezoidal asymmetrical flow field-flow fractionation channels. Anal. Chem..

[46] FDA/CDER/CBER, Drug Products (2017). Including biological products. That Contain NanomaterialsGuidance for Industry in.

