# Supplementary material for: Physical characterization of liposomal drug formulations using multi-detector asymmetrical-flow field flow fractionation
Source: J Control Release. 2020 Apr 10;320:495–510. doi: 10.1016/j.jconrel.2020.01.049 (PMC7146538; doi:10.1016/j.jconrel.2020.01.049)
Supplement: Supplementary file 1 — Supplementary material [file mmc1.docx]

# Supplemental Information

Physical characterization of liposomal drug formulations using multi-detector asymmetrical-flow field flow fractionation

Parot, J.^a,b^, Caputo, F.^c^, Mehn, D.^d^, Hackley, V.A.^a^, Calzolai, L.^d^

^a^ Materials Measurement Science Division, National Institute of Standards and Technology, Gaithersburg, Maryland 20899-8520, USA

^b^ Theiss Research, La Jolla, California 92037, USA

^c^ Université Grenoble Alpes, CEA, LETI, F-38000 Grenoble, France

^d^ European Commission, Joint Research Centre (JRC), Ispra, Italy

## 1. Instrumental configurations by laboratory

**Lab 1:** Agilent Technologies (Santa Clara, CA USA)^[[1]](#footnote-1)^ 1100-series isocratic pump equipped with a Gastorr TG-14 degasser, fan Agilent 1260 ALS autosampler with a 900 μL injection loop connected to a Wyatt Technology Eclipse DualTec AF4 system. On-line detectors include an Agilent 1200 UV-VIS absorbance diode array detector with spectral range (190 to 950) nm (ii) a Wyatt DAWN HELEOS II MALS detector (λ=661 nm) equipped with 18 detectors at angles from 12.8° to 157.8° (table S3), a standard flow cell (1.2mm diameter), a fiber optic based Wyatt on-line quasi-elastic light scattering (QELS) DLS detector installed at a scattering angle of 99.9° (MALS position LS-12) and/or (iii) a Zetasizer Nano ZS (Malvern Panalytical) with a wavelength of 633 nm and operating in flow mode at a scattering angle of 177°. Data analysis was performed with Wyatt ASTRA Version 6.1.7.17 and Zetasizer software for flow DLS.

**Lab 2:** Agilent 1260-series Infinity Bio-inert Quaternary Pump equipped with an Agilent 1260 Infinity Bio-inert High Performance Autosampler (100µL injection loop) connected to a Wyatt Eclipse AF4 system. On-line detectors include (i) an Agilent 1200 UV-VIS absorbance detector with single wavelength selectable from (190 to 800) nm and (ii) a Wyatt DAWN HELEOS II MALS detector (λ= 664.3 nm), a standard flow cell (1.2mm diameter) and with a Wyatt QELS installed at a scattering angle of 134° (MALS position LS-16). Data analysis was performed with Wyatt ASTRA Version 6.1.2.84.

**Lab 3:** Postnova AF2000 AF4 system (Postnova Analytics GmbH, Landsberg, Germany) equipped with degasser (Postnova 7520), two isocratic pumps (Postnova 1130), using a manual injection loop of 100 μL. The system uses the following on-line detectors: (i) a UV-VIS detector (Postnova 3212) with single wavelength selectable from 190 nm to 800 nm; (ii) a Postnova 3621 MALS detector (λ=532 nm) equipped with 21 detectors at angles from 7° to 164° (table S3); (iii) Zetasizer Nano ZS (Malvern Panalytical) equipped with flow-mode cell, using a wavelength of 633 nm and detecting in backscattering at 173°. Data analysis was performed with Postnova data analysis software 2.1.0.5. (AF4-UV-MALS data) and Zetasizer software for flow DLS.

## 2. Data analysis and reporting

Fractogram presentation

Fractograms are reported by plotting the UV-Vis and/or light scattering intensity at a specific angle (e.g., 90° for MALS, 173° for the Zetasizer) versus the elution time beginning when focus/relaxation ends and detector flow (with or without cross flow) begins. Normalized intensities are extracted as processed by the instrumental software. More detailed information on the light scattering properties of the samples can be obtained in specific situations (e.g., by extracting the unnormalized excess Rayleigh ratio from each MALS angle). Mass recovery, retention time, void time and the retention ratio are obtained using the fractogram trace from the concentration detector (UV-Vis at 280 nm in the method presented here). Retention and void time are determined at the peak maximum. The analyst should report recovery, retention time, void time and retention ratio, in addition to recovery.

Particle size determination

For the purpose of this optimized MD-AF4 method, the Berry model for analysis of MALS data (to obtain the rms radius, *R*_g_) is recommended for the general analysis of liposomal samples, being one of the suggested models to be used over a wide size range (without specific size limitations) when the shape of the particle is not known (no spherical assumption is necessary).

Berry plot: $\sqrt{\frac{Kc}{R_{\theta}}}$ vs. ${sin}^{2}\left( \frac{\theta}{2} \right)$

where *K* is an optical constant, *c* is the mass concentration of scatterers, *R*_θ_ is the excess Rayleigh ratio, and θ is the scattering angle. To obtain *R*_g_ only (not molar mass) the Berry plot does not require knowledge of the concentration. Since different models can generate somewhat different results, the approach used in the data analysis should always be reported. MALS data should be analyzed selecting a model based on the knowledge of the analyte (size range, shape if known) and quality of the fit (e.g., by evaluating the fit *R*^2^ value across the peak), and by adjusting the number of MALS angles used in the model based on quality of fit and quality of data at each angle. Based on our experience, we recommend using a minimum of 8 angles for analysis.

The use of a DLS detector provides hydrodynamic size, *R*_h_, during fractionation. For the purpose of this optimized MD-AF4 method, DLS data should be analyzed according to the quality of the fit (e.g., by evaluating the fit error across the peak), the correlation function shape/noise and by adjusting the processing parameters as required. Note that different processes are available to analyze measured correlation data obtained online. For instance, a single exponential fit versus cumulant analysis. The Zetasizer in flow-mode operation uses cumulants analysis, while the QELS detector provides several methods including cumulants and single exponential. In any case, the analysis method should be reported. Theoretically, the single exponential and cumulants analysis should produce similar results for online DLS, since each data slice in the fractogram is essentially monodisperse.

Reporting particle size

In order to calculate and report an average size value (*R*_g_ and/or *R*_h_), ISO/TS 21362:2018 suggests that, when a monodispersed or a monomodal sample characterized by a limited polydispersity is measured and a symmetric elution peak is obtained, the mean size value should be calculated by averaging the values across the full width at half maximum (FWHM) (arithmetic mean). On the contrary, when the sample is characterized by a clear upward trend and/or by multiple populations which are not completely resolved, the calculated mean across the FWHM might not represent the true mean size of the particle population. In a similar scenario, we suggest only to plot the size values across the FWHM in the fractogram and to report the mode (peak maximum) values of the detectable peaks.

For all samples the spread value (difference between the minimum and maximum value across the FWHM) can be an indication of the polydispersity of the sample.

The shape factor ($\rho={R_{g}}/{R_{h}})$ should be reported in a manner similar to that described above for size values.

## 3. AF4 PSL elution method

The method used to analyze NIST Traceable PSL spheres consisted of a focus-injection step for 5 min at focus flow 1, followed by elution at detector flow DF=(0.5 or 1) mL/min with an exponentially decaying cross flow from XF=(1 to 0) mL/min for 45 min. The trapezoidal long channel (Wyatt) was equipped with a 10 kDa PES membrane and a 350 µm spacer. The mobile phase was 0.5 mmol/L ammonium nitrate and the injection volume was 10 μL.

| **Step duration (min)** | **Mode** | **XF (mL/min)** |
| --- | --- | --- |
| 2 | Elution | 0 |
| 2 | Focus | - |
| 5 | Focus + Inject | - |
| 45 | Elution | 1 to 0 |
| 10 | Elution | 0 |

## 4. AF4 between sample washing protocol

100 µL of PBS is injected during a focus-injection step for 8 min at focus flow rate of 2 mL/min, followed by elution at detector flow DF= 0.5 mL/min without cross flow for 13 min. This procedure is repeated after each sample analysis to help ensure that memory effects are minimized and that each sample injection yields consistent results.

| **Step duration (min)** | **Mode** | **cross flow (mL/min)** |
| --- | --- | --- |
| 2 | Elution | 0 |
| 2 | Focus | - |
| 8 | Focus + Inject | - |
| 13 | Elution | 0 |

## 5. Supplementary figures

Note: all flow rates given in mL/min, XF=cross flow, DF=detector flow.

Figure S1: Test of multiple elution programs for Dox1. *AF4 fractograms showing normalized excess Rayleigh ratio at 90° versus elution time with R_g_ overlaid. Injection of 50 µg Dox1 and applying A) constant cross flow with XF=0.3 or 0.5, two stage cross flow with XF=1 and 0.5 then 1 and 0.3, exponential cross flow decay, B) linear cross flow decay to XF=0 in 45 min (starting XF=0.8, 1, 1.25), C) linear cross flow decay to XF=0 in 30, 45 or 60 min (starting XF= 1 and DF=0.5).*

Figure S2: Comparison of three elution methods and DF for Dox1. *AF4 fractograms showing normalized excess Rayleigh ratio at 90° versus elution time with R_g_ (lines) and R_h_ (dots) overlaid. Injection of 50 µg of Dox1 and applying a constant cross flow XF=0.3 (black), or an exponential decay from XF=1 to XF=0 in 60 min (purple) or a linear decay from XF=1 to XF=0 in 30 min (green) with a DF of A) 0.5 or B) 1.*

Figure S3: *Measurement of Dox1 using different sizing detectors as measured by Lab1. DF=0.5 (black) and DF=1 (orange). A) AF4 fractograms showing normalized excess Rayleigh ratio at 90° and R_g_ measured by MALS, B) AF4 fractogram and R_h_ measured by online DLS (Wyatt QELS) and C) AF4 fractogram (normalized kcps) and hydrodynamic radius R_h_ measured by online DLS (Malvern Zetasizer).*

Figure S4: Test of different conditions for Dox2. *AF4 fractograms showing normalized excess Rayleigh ratio at 90° versus elution time with R_g_ (lines) and R_h_ (dots) for 25 µg injection of Dox2. A) comparison of elution method by applying a constant cross flow XF=0.3 (black), or an exponential decay from XF=1 to XF=0 in 60 min (purple) or a linear decay from XF=1 to XF=0 in 30 min (red). B) effect of channel spacer (black: 250 µm, violet: 350 µm, green: 490 µm) and C) effect of focus time (black: 3 min, purple: 5 min, red: 8 min).*

Figure S5: Effect of injected mass for Dox2*. Injection of (5 to 200) µg applying the optimized method. A) AF4 fractograms showing normalized excess Rayleigh ratio at 90° versus elution time with R_g_ overlaid, B) fractograms with R_h_ (dots) measured by online QELS overlaid, and C) UV-Vis response reporting the area under the eluted peaks as a function of injected mass.*

Figure S6: *Repeatability of the optimized method by injecting 3 or more replicate samples. A) Dox1, B) Dox1C, C) Dox2, D) Dox3 E) Dox 4, and F) Cipro. AF4 fractograms showing* *normalized excess Rayleigh ratio at 90° versus elution time with R_g_ (line) and R_h_ (dots) overlaid.*

Figure S7: *Comparison of AF4 fractograms showing normalized excess Rayleigh ratio at 90° versus elution time with R_g_ (lines) and R_h_ (dots) overlaid, for Dox1 (red) and its control Dox1C (black) using the optimized method.*

## 6. Supplementary tabular data

Table S1: Composition and pH of mobile phases used in this study. Concentration is reported in g/L. Hydrates are converted to equivalent anhydrous content.

| Buffer code | Buffer description | Product number | NaCl | KCl | Na_2_HPO4 | KH_2_PO4 | pH^[[2]](#footnote-2)^ | Experiments performed/Lab |
| --- | --- | --- | --- | --- | --- | --- | --- | --- |
| PBS1 | PBS from Lonza | 17-516Q | 9 | 0 | 0.421 | 0.144 | 7.4 ± 0.1 | Lab 1: method optimization |
| PBS2 | DPBS from Lonza | 17-512F | 8 | 0.2 | 1.144 | 0.2 | 7.3 ± 0.1 | Lab 1: method optimization  Lab 2: method optimization and analysis of Dox1 with the optimized method |
| PBS3 | PBS from GE Hyclone Classic | 16777-252 | 8 | 0.2 | 1.15 | 0.2 | 7.2 ± 0.1 | Lab 1: method optimization and analysis of the different formulations with the optimized method |
| PBS4 | PBS (tablets) from Gibco, ThermoFisher | 10010031 | 8.12 | 0.201 | 0.95 | **-** | 7.4 ± 0.1 | Lab 3: analysis of Dox1 using the optimized method |
| Saline | NaCl 0.9% VWR Chemicals BDH | BDH7257-1 | 9 | 0 | 0 | - | 6.0 ± 0.1 | Lab 1: method optimization |

Table S2: Description of liposomal formulations used in this study as provided by the vendor. Z-avg is the intensity weighted hydrodynamic diameter calculated using cumulants analysis of DLS data. PI is the polydispersity index obtained from cumulants analysis.

| **Sample code** | **Sample description** | **Size (r.nm) and PI** | **Total API** | **Total lipid content (mg/mL)** | **Provider** |
| --- | --- | --- | --- | --- | --- |
| **Dox1** | Research grade commercial liposomal doxorubicin hydrochloride product with same P-C properties as RLD Doxil in isotonic sucrose with histidine buffer. | Z-avg= 78 nm PI= 0.06 | Doxorubicin HCl at 2.0 mg/mL | 15.8 | Lipocure LtD batch #101071 |
| **Dox1C** | Research grade empty control liposomes. In isotonic sucrose with histidine buffer. | Z-avg=78 nm PI= 0.04 | 0 | 15.6 | Lipocure LtD batch #500010 |
| **Dox2** | Research grade liposomal doxorubicin hydrochloride. Polymodal and polydisperse product. Contains 10 % sucrose and histidine buffer. Stored frozen. | Z-avg= 135 nm PI= na | Doxorubicin HCl at 2.2 mg/mL | 26.8 | Avanti polar Product code 300115, lot #300115-01-010 |
| **Dox3** | Pharmaceutical grade liposomal doxorubicin hydrochloride (generic Doxil) in sucrose with histidine buffer. | na | Doxorubicin HCl at 2.0 mg/mL  (<10% free) | 15.9 | Dr Reddy Laboratories LTD NDC 43598-283-35 |
| **Dox4** | Pharmaceutical grade liposomal doxorubicin hydrochloride product (generic Doxil) in sucrose with histidine buffer. | na | Doxorubicin HCl at 2.0 mg/mL  (<10% free) | 15.9 | Sun Pharmaceutical Industries LTD  NDC 47335-050-40 |
| **Cipro** | Research grade liposomal ciprofloxacin. | Z-avg= 80 nm, PI=0.1 | Ciprofloxacin at 1 mg/mL  (<5% free) | 20 | ProFoldin Lot PHPC002CP |

na=not available

Table S3: MALS angles and positions for Wyatt DAWN HELEOS II MALS and Postnova 3621 MALS

| LS Position | Fixed detector angles | |
| --- | --- | --- |
|  | Wyatt DAWN HELEOS II MALS | Postnova 3621 MALS |
| 1 | 22.5 | 7 |
| 2 | 28 | 12 |
| 3 | 32 | 20 |
| 4 | 38 | 28 |
| 5 | 44 | 36 |
| 6 | 50 | 44 |
| 7 | 57 | 52 |
| 8 | 64 | 60 |
| 9 | 72 | 68 |
| 10 | 81 | 76 |
| 11 | 90 | 84 |
| 12 | 99 | 90 |
| 13 | 108 | 100 |
| 14 | 117 | 108 |
| 15 | 126 | 116 |
| 16 | 134 | 124 |
| 17 | 141 | 132 |
| 18 | 147 | 140 |
| 19 |  | 148 |
| 20 |  | 156 |
| 21 |  | 164 |

Table S4: Summary of results obtained by batch mode (off-line) DLS using cumulants analysis (mean hydrodynamic radius (*R*_h_) and polydispersity index (PI)), and the intensity-weighted mean hydrodynamic radius calculated by non-negative constrained least squares analysis (Peak 1). Mean of 5 measurements reported. SD = standard deviation of the mean.

| Sample Name | *R*_h_ (SD) | PI (SD) | Peak 1 (SD) |
| --- | --- | --- | --- |
|  | r.nm |  | r.nm |
| Dox1 | 40 (1) | 0.06 (0.02) | 42.5 (2) |
| Dox2 | 56 (2) | 0.26 (0.02) | 73.5 (9) |
| Dox3 | 41 (1) | 0.03 (0.02) | 43 (1) |
| Dox4 | 39 (1) | 0.05 (0.01) | 41.5 (1) |
| Cipro | 43.5 (1) | 0.01 (0.02) | 48.5 (4) |

Table S5: Summary of results obtained for analysis of Dox1 and applying multiple elution programs at a detector flow DF=0.5 mL/min. Recovery, retention time and retention ratio were measured at an absorbance of 280 nm. *R*_g_ was determined using the Berry model with MALS data and *R*_h_ were averaged across the FWHM by QELS (Wyatt) at the angle positions 134°. Only one injection for each condition was tested. na=not applicable

| Elution programs | DF (mL/min) | Recovery (%) | Retention  time (min) | Retention  ratio | *R*_g_ (nm) | *R*_g_ Spread (nm) | *R*_h_ (nm) | *R*_h_ Spread (nm) | *R*_g_/*R*_h_ |
| --- | --- | --- | --- | --- | --- | --- | --- | --- | --- |
| Constant XF 0.3 | 0.5 | 94 | 13.5 | 0.018 | 29.9 | 10.3 | na | na | na |
| Constant XF 0.5 | 0.5 | 96 | 24.2 | 0.010 | 28.5 | 14.9 | na | na | na |
| Constant XF1=1 (10 min) XF2=0.5 | 0.5 | 96 | 31.1 | 0.008 | 29.3 | 11.8 | na | na | na |
| Constant XF1=1 (10 min) XF2=0.3 | 0.5 | 94 | 22.3 | 0.011 | 29.6 | 11.2 | na | na | na |
| Exponential 1 => 0 (45min) | 0.5 | 94 | 19.3 | 0.013 | 29.3 | 11.9 | na | na | na |
| Linear 1 => 0 (45min) | 0.5 | 94 | 31.3 | 0.008 | 30.1 | 11.0 | na | na | na |
| Linear 0.8 => 0 (45min) | 0.5 | 94 | 27.4 | 0.009 | 29.5 | 11.8 | na | na | na |
| Linear 1.25 => 0 (45min) | 0.5 | 94 | 35.5 | 0.007 | 30.1 | 11.4 | na | na | na |
| Linear 1 => 0 (30min) | 0.5 | 98 | 23.4 | 0.010 | 30.9 | 11.5 | 34.6 | 9.7 | 0.89 |
| Linear XF1 => 0 (60 min) | 0.5 | 95 | 31.3 | 0.008 | 30.2 | 12.2 | 35.8 | 11.2 | 0.84 |

Additional measurement conditions: Membrane RC 10 kDa, Long channel, mass injected=50 µg, sample diluted at 1 mg/mL in the elution buffer (DPBS by Lonza), focus flow of 2 mL/min for 8 min.

Table S6: Summary of results obtained for analysis of Dox1 and applying multiple elution programs at a detector flow DF=1 mL/min. Recovery, retention time and retention ratio were measured at an absorbance of 280 nm. *R*_g_ was determined using the Berry model. *R*_g_ and *R*_h_ were averaged across the FWHM by QELS (Wyatt) at the angle positions 134°. Only one injection for each condition was tested.

| Elution programs | DF (mL/min) | Recovery (%) | Retention  time (min) | Retention  ratio | *R*_g_ (nm) | *R*_g_ Spread (nm) | *R*_h_ (nm) | *R*_h_ Spread (nm) | *R*_g_/*R*_h_ |
| --- | --- | --- | --- | --- | --- | --- | --- | --- | --- |
| Constant XF 0.3 | 1 | 96 | 7.3 | 0.016 | 30.3 | 10.5 | 32.7 | 7.8 | 0.93 |
| Constant XF 0.5 | 1 | 96 | 11.8 | 0.010 | 30.5 | 13.7 | 33.5 | 10.8 | 0.91 |
| Constant XF1=1 (10 min) XF2=0.5 | 1 | 95 | 16.5 | 0.007 | 30.4 | 14.6 | 33.7 | 10.1 | 0.90 |
| Linear 1 => 0 (45min) | 1 | 95 | 18.5 | 0.006 | 31.5 | 14.8 | 34.4 | 10.7 | 0.92 |
| Linear 0.8 => 0 (45min) | 1 | 97 | 15.6 | 0.008 | 31.0 | 14.4 | 33.9 | 10.3 | 0.91 |
| Linear 1.25 => 0 (45min) | 1 | 97 | 22.0 | 0.005 | 31.6 | 14.9 | 33.6 | 10.6 | 0.93 |
| Linear 1 => 0 (30min) | 1 | 96 | 17.5 | 0.007 | 30.8 | 11.7 | 33.3 | 7.4 | 0.92 |
| Linear 1 => 0 (60 min) | 1 | 95 | 21.4 | 0.005 | 30.7 | 12.4 | 33.7 | 7.5 | 0.91 |

Additional measurement conditions: Membrane RC 10 kDa. Long channel, mass injected 50 µg, sample diluted at 1 mg/mL in the elution buffer (DPBS from Lonza), focus flow of 2 mL/min for 8 min.

Table S7: Summary of results obtained by injecting Dox1 and applying multiple elution programs at a detector flow rate DF=0.5 or 1 mL/min. Recovery, retention time and retention ratio were measured at an absorbance of 280 nm. The average and spread of *R*_g_ (Berry model) and *R*_h_ were calculated across the FWHM of the intensity peak by QELS (Wyatt) at the angle positions 134°. The average and standard deviation calculated for 3 replicate injections are reported.

| Elution programs | DF (mL/min) | Recovery (%) | Retention  time (min) | Retention  ratio | *R*_g_ (nm) | *R*_g_ Spread (nm) | *R*_h_ (nm) | *R*_h_ Spread (nm) | *R*_g_/*R*_h_ |
| --- | --- | --- | --- | --- | --- | --- | --- | --- | --- |
| Constant XF 0.3 | 0.5 | 95 (1) | 13.1 (0.5) | 0.019 (0.001) | 30.2 (0.1) | 11.8 (0.2) | 35.9 (0.1) | 11.3 (0.4) | 0.83 (0.03) |
| Exponential XF1->0 (60 min) | 0.5 | 93 (0.3) | 22.4 (0.1) | 0.011 (0.001) | 31.2 (0.1) | 13.3 (0.1) | 36.5 (0.1) | 12.2 (0.9) | 0.85 (0.03) |
| Gradient XF1->0 (30 min) | 0.5 | 95 (0.7) | 18.1 (0.1) | 0.014 (0.001) | 29.9 (0.1) | 12.1 (0.1) | 35.9 (0.3) | 11 (1) | 0.83 (0.08) |
| Constant XF 0.3 | 1 | 96 (0.1) | 7.2 (0.1) | 0.016 (0.001) | 30.2 (0.1) | 10.4 (0.1) | 33.2 (0.1) | 7.6 (0.6) | 0.91 (0.03) |
| Exponential XF1->0 (60 min) | 1 | 96 (0.1) | 13.0 (0.1) | 0.009 (0.001) | 30.2 (0.4) | 13.1 (0.8) | 33.6 (0.1) | 9.6 (0.5) | 0.89 (0.01) |
| Linear XF1->0 (30 min) | 1 | 97 (0.1) | 16.3 (0.1) | 0.007 (0.001) | 30 (1) | 13.3 (0.1) | 33.9 (0.1) | 9.4 (0.1) | 0.89 (0.03) |

Additional measurement conditions: Membrane RC 10 kDa. Long channel, mass injected 25 µg, sample diluted at 1 mg/mL in the elution buffer (DPBS from Lonza), focus 2 mL/min for 8 min.

Table S8: Summary of results obtained by injecting the control Dox1C and applying multiple elution programs at a detector flow rate DF=0.5 or 1 mL/min. Recovery, retention time and retention ratio were measured at an absorbance of 280 nm. The average and spread of *R*_g_ (Berry model) and *R*_h_ were calculated across the FWHM of the intensity peak by QELS (Wyatt) at the angle positions 134°. The average and standard deviation calculated for 3 replicate injections are reported.

| Elution programs | DF (mL/min) | Recovery (%) | Retention  time (min) | Retention  ratio | *R*_g_ (nm) | *R*_g_ Spread (nm) | *R*_h_ (nm) | *R*_h_ Spread (nm) | *R*_g_/*R*_h_ |
| --- | --- | --- | --- | --- | --- | --- | --- | --- | --- |
| Constant XF 0.3 | 0.5 | 92 (0.4) | 13.2 (0.02) | 0.019 (0.001) | 32.1 (0.1) | 11.9 (0.07) | 36.4 (0.1) | 10.8 (0.1) | 0.88 (0.01) |
| Exponential XF1->0 (60 min) | 0.5 | 92 (0.8) | 18.4 (0.05) | 0.014 (0.001) | 32.5 (0.1) | 12.5 (0.1) | 36.4 (0.1) | 11.4 (0.2) | 0.89 (0.02) |
| Linear XF1->0 (30 min) | 0.5 | 90 (0.5) | 22.9 (0.1) | 0.011 (0.001) | 32.2 (0.4) | 13.2 (0.02) | 37.0 (0.1) | 12.3 (0.2) | 0.87 (0.01) |
| Constant XF 0.3 | 1 | 96 (0.1) | 7.5 (0.05) | 0.016 (0.001) | 32.2 (0.5) | 10 (1) | 33.4 (0.2) | 8 (1) | 0.96 (0.01) |
| Exponential XF1->0 (60 min) | 1 | 97 (0.1) | 13.4 (0.1) | 0.0089 (0.007) | 32.3 (0.2) | 12.9 (0.1) | 33.3 (0.7) | 9.7 (0.7) | 0.97 (0.02) |
| Linear XF1->0 (30 min) | 1 | 94 (0.1) | 16.7 (0.04) | 0.007 (0.002) | 31.8 (0.1) | 13.2 (0.07) | 33.8 (0.4) | 8.4 (0.9) | 0.94 (0.01) |

Additional measurement conditions: Membrane RC 10 kDa. Long channel, mass injected 25 µg, sample diluted at 1 mg/mL in the elution buffer (DPBS from Lonza), focus 2 mL/min for 8 min.

Table S9: Summary of results obtained measuring the size of NIST Traceable PSL spheres in the range (15-175) nm (nominal radius) and Dox1 using multiple online sizing detectors: Wyatt MALS and online QELS (mounted in position LS-12 (99.9º) or LS-16 (134º)) and Malvern Zetasizer (173º) operated in flow-mode as the final detector. *R_s_* obtained by MALS using the sphere form factor for PSL and *R*_g_ determined using the Berry form of the Debye model for Dox1. The hydrodynamic radius (from QELS or Z-Avg/2 from the Zetasizer) are compared to the stated size values at two different detector flow rates (DF=0.5 and DF=1). Mean (standard deviation) are calculated across the FWHM of the fractograms peak.

|  |  | DF 0.5 | | | DF 1 | | |  |
| --- | --- | --- | --- | --- | --- | --- | --- | --- |
| Sample | QELS angle | MALS | QELS | Zetasizer | MALS | QELS | Zetasizer | Stated radius |
| r.nm. |  | ***R_s_* or *R*_g_ (nm)** | ***R*_h_ (nm)** | ***z-avg*/2 (nm)** | ***R_s_* or *R*_g_ (nm)** | ***R*_h_ (nm)** | ***z-avg*/2 (nm)** | ***R* (nm)** |
| PSL 15 | 99.9º | 14 (1) | 15 (1) | 17.5 (2) | 16 (1) | 14.5 (1) | 17 (1.5) | 15.5 ± 3 |
|  | 134º | - | - | - | - | - | - | - |
| PSL 30 | 99.9º | 29.5 (1) | 26.5 (1) | 29 (1.5) | 30 (1) | 24 (1) | 30.5 (1) | 30.5 ± 4 |
|  | 134º | - | 30.5 (1) | - | - | 32 (2) | - | - |
| PSL 62.5 | 99.9º | 62.5 (1) | - | - | 62 (1) | - | - | 62.5 ± 3 |
|  | 134º | - | 61 (2) | - | - | 54 (4) | - | - |
| PSL 75 | 99.9º | 71.5 (0.5) | 51.5 (2) | 68.5 (3.5) | 31 (0.5) | - | 67.5 (1) | 75 ± 4 |
|  | 134º | - | - | - |  | 38 (2) | - | - |
| PSL 100 | 99.9º | 101.1 (0.5) | 62.5 (5) | 93.5 (3) | 100.5 (0.5) | 53 (4) | 102 (3.5) | 102 ± 6 |
|  | 134º | - | 105 (7.5) | - | - | 89 (9) | - | - |
| PSL 175 | 99.9º | 184 (1.5) | - | - | 181 (2) | - | - | 175 ± 6 |
|  | 134º | - | 166 (30) | - | - | 258 (42) | - | - |
| Dox1 | 99.9º | 29.4 | 35 | 41.5 | 14.3 | 13.9 | 46.5 | - |
|  | 134º | - | 36 | - | - | 16.5 | - | - |

Table S10: Summary of results obtained for Dox1 in the long channel applying a detector flow DF=0.5 or 1 mL/min. *R*_h_ measured by QELS (Wyatt) at the angle positions 134 and 99°. In the table the recovery, retention time and retention ration were measured at an absorbance of 280 nm. The average and spread of *R*_g_ (Berry model) and *R*_h_ (single exponential fit) calculated across the FWHM of the peak are reported. Only one injection was conducted for each condition, except where specified. *Average (standard deviation) reported for 5 replicate measurements.

| Sample | DF (mL/min) | DLS angle | Recovery (%) | Retention  time (min) | Retention  ratio | *R*_g_ (nm) | *R*_g_ Spread (nm) | *R*_h_ (nm) | *R*_h_ Spread (nm) | *R*_g_/*R*_h_ |
| --- | --- | --- | --- | --- | --- | --- | --- | --- | --- | --- |
| Dox1* | 0.5 | 134º | 97 | 12.0 (0.1) | 0.04 (0.03) | 28.7 (0.3) | 12.1 (0.2) | 34.2 (0.5) | 10.9 (1.5) | 0.84 (0.02) |
| Dox1* | 0.5 | 99.9º | 98 (1) | 11.9 (0.3) | 0.04 (0.03) | 28.9 (0.3) | 11.9 (0.6) | 34.2 (0.4) | 11.0 (1.4) | 0.84 (0.02) |
| Dox1 | 1 | 134º | 95 | 7.6 | 0.02 | 28.7 | 10.9 | 28.9 | 5.5 | 0.99 |
| Dox1 | 1 | 99.9º | 96.2 (0.1) | 7.2 (0.1) | 0.01678 (0.0005) | 30.2 (0.1) | 10.4 (0.1) | 33.2 (0.1) | 7.6 (0.6) | 0.91(0.03) |

Additional measurement conditions: constant cross flow XF=0.3 mL/min, injected mass 25 µg, long channel equipped with a 10 kDa RC membrane, applying a focus time of 8 min and a focus flow of 2 mL/min, sample diluted to 1 mg/mL in the elution buffer (PBS from Hyclone).

Table S11: Summary of the results obtained for Dox1 and Dox1C in the long and in the short channel. *R*_h_ measured by QELS (Wyatt) at the angle position 99°. Recovery, retention time and retention ratio measured at an absorbance of 280 nm. The average and spread of *R*_g_ (Berry model) and *R*_h_ (single exponential fit) were calculated across the FWHM of the peak. Only one injection was conducted for each condition, except where specified. *Average (SD) measured over 5 measurements.

| Sample | Channel | Recovery (%) | Retention  time (min) | Retention  ratio | *R*_g_ (nm) | *R*_g_ Spread (nm) | *R*_h_ (nm) | *R*_h_ Spread (nm) | *R*_g_/*R*_h_ |
| --- | --- | --- | --- | --- | --- | --- | --- | --- | --- |
| Dox1 | SC | 98 | 15.2 | 0.03 | 28.7 | 14.4 | 34.0 | 11.2 | 0.84 |
| Dox1* | LC | 98 (1) | 11.9 (0.3) | 0.04 (0.03) | 28.9 (0.3) | 11.9 (0.6) | 34.2 (0.4) | 11.0 (1.4) | 0.84 (0.02) |
| Dox1C | SC | 102 | 15.9 | 0.02 | 31.0 | 14.6 | 34.5 | 11.0 | 0.89 |
| Dox1C | LC | 110 | 12.8 | 0.03 | 28.7 | 13.1 | 34.0 | 10.9 | 0.84 |

Additional measurement conditions: constant cross flow XF=0.3 at detector flow DF=0.5 mL/min, injected mass 25 µg, channel equipped with a 10 kDa RC membrane, applying a focus time of 8 min, and a focus flow of 2 mL/min, sample diluted to 1 mg/mL in the elution buffer (PBS from Hyclone).

Table S12: Summary of results obtained for Dox1 and Dox2 in short channel equipped with spacers of 250 µm, 350 µm and 490 µm. *R*_h_ measured by a QELS (Wyatt) at the angle position 99°. Recovery, retention time and retention ratio were measured at an absorbance of 280 nm. The average and spread of *R*_g_ (Berry model) and *R*_h_ (single exponential fit) were calculated across the FWHM of the peak. Only one injection for each condition was tested, except where specified. *Average (standard deviation) reported for 5 replicate measurements. In the case of Dox2, which is composed of multiple populations, the size value calculated at the LS 90° peak maximum are also reported.

| Sample | Spacer | Recovery (%) | Retention time (min) | Retention ratio | *R*_g_ (nm) | *R*_g_ Spread (nm) | *R*_h_ (nm) | *R*_h_ Spread (nm) | *R*_g_/*R*_h_ |
| --- | --- | --- | --- | --- | --- | --- | --- | --- | --- |
| Dox1 | 250 µm | 110 | 6.0 | 0.06 | 29.5 | 10.8 | 31.5 | 7 | 0.94 |
| Dox1* | 350 µm | 98 (1) | 11.9 (0.3) | 0.04 (0.03) | 28.9 (0.3) | 11.9 (0.6) | 34.2 (0.4) | 11.0 (1.4) | 0.84 (0.02) |
| Dox1 | 490 µm | 101 | 32.7 | 0.01 | 29.8 | 13.2 | 33.1 | 8.1 | 0.900 |
| Dox2 | 250 µm | 65 | Peak 1: 4.5  Peak 2:  7.00 | Peak 1: 0.075  Peak 2: 0.05 | Peak 1^#^: 24.5  Peak 2^#^: 37.3 | FWHM: 55.3 | Peak 1*: 28.0  Peak 2*: 36.1 | FWHM: 44.6 | Peak 1^#^: 0.88  Peak 2^#^: 1.03 |
| Dox2* | 350 µm | 93 (6) | Peak 1: 8.6 (0.2)  Peak 2: 15.0 (0.7) | Peak 1: 0.048 (0.008)  Peak 2: 0.027 (0.005) | Peak 1: 20 (2)  Peak 2: 35.5 (0.2) | FWHM: 61 (1) | Peak 1:  26 (2)  Peak 2: 39 (1) | FWHM: 76 (16) | Peak 1: 0.76 (0.03)  Peak 2: 0.90 (0.03)  FWHM: 0.72 (0.05) |
| Dox2 | 490 µm | 62 | Peak 1: 21.7  Peak 2: 40.7 | Peak 1: 0.02  Peak 2: 0.01 | Peak 1: 15.7  Peak 2: 36.2 | FWHM:40 | Peak 1:  24.0  Peak 2: 35.5 | FWHM: 42 | Peak 1: 0.67  Peak 2: 1.01 |

Additional measurement conditions: constant cross flow XF=0.3 at detector flow DF=0.5 mL/min, injected mass 25µg, short channel equipped with a 10 kDa RC membrane, applying a focus time of 8 min, and a focus flow of 2 mL/min, sample diluted to 1 mg/mL in the elution buffer (PBS from Hyclone).

Table S13: Test of multiple membranes. Summary of the results obtained by using (i) 10 kDa and 30 kDa membrane of RC and focus time of 8 min or (ii) 10 kDa membrane of RC and PES and focus time of 5 min. *R*_h_ measured by QELS (Wyatt) at the angle position 99°. Recovery, retention time and retention ratio were measured at an absorbance of 280 nm. The average and spread of *R*_g_ (Berry model) and *R*_h_ (single exponential fit) were determined across the FWHM of the peak. Only one injection for each condition was tested.

| Sample | Membrane | Recovery (%) | Retention time (min) | Retention ratio | *R*_g_ (nm) | *R*_g_ Spread (nm) | *R*_h_ (nm) | *R*_h_ Spread (nm) | *R*_g_/*R*_h_ |
| --- | --- | --- | --- | --- | --- | --- | --- | --- | --- |
| Dox1 | 10 kDa RC | 98 | 15.2 | 0.03 | 28.7 | 14.4 | 34.0 | 11.2 | 0.84 |
| Dox1 | 30 kDa RC | 96 | 10.5 | 0.03 | 26.7 | 13.5 | 34.0 | 9.3 | 0.78 |
| Dox1 | 10kDa PES* | 84 | 17.6 | 0.03 | 27.8 | 14.2 | 34.7 | 12.6 | 0.80 |
| Dox1C | 10 kDa RC | 102 | 15.9 | 0.02 | 31.0 | 14.6 | 34.5 | 11.0 | 0.89 |
| Dox1C | 30 kDa RC | 99 | 11.0 | 0.05 | 28.3 | 11.0 | 34.7 | 12.9 | 0.81 |
| Dox1C | 10kDa PES* | 70 | 21.6 | 0.02 | 28.5 | 13.9 | 35.0 | 11.3 | 0.81 |

*measured using the long channel.

Additional measurement conditions: constant cross flow XF=0.3 at detector flow DF=0.5 mL/min, injected mass 25 µg, long channel equipped with a 350 µm spacer, applying a focus time of 8 min, and a focus flow of 2 mL/min, sample diluted to 1 mg/mL in the elution buffer (PBS from Hyclone).

Table S14: Summary of results for Dox1 using different mobile phases (PBS from Lonza and Hyclone, DPBS from Lonza, NaCl 0.9%). *R*_h_ measured by QELS (Wyatt) at the angle position 99°. Recovery, retention time and retention ratio were measured at an absorbance of 280 nm. The average and spread of *R*_g_ (Berry model) and *R*_h_ (single exponential fit) were determined across the FWHM of the peak.

| Sample | Elution Buffer | Recovery (%) | Retention  time (min) | Retention ratio | *R*_g_ (nm) | *R*_g_ Spread (nm) | *R*_h_ (nm) | *R*_h_ Spread (nm) | *R*_g_/*R*_h_ |
| --- | --- | --- | --- | --- | --- | --- | --- | --- | --- |
| Dox1 | PBS Lonza | 108 (1) | 12.7 (0.01) | 0.027 | 29.7 (0.1) | 11.6 (0.1) | 32.3 (0.1) | 8.3 (0.5) | 0.91 (0.01) |
| Dox1 | DBPS Lonza | 106 (1) | 13.4 (0.1) | 0.021 | 30.0 (0.1) | 11.6 (0.1) | 32.7 (0.1) | 9.3 | 0.92 (0.01) |
| Dox1 | PBS Hyclone | 101 | 12.9 | 0.027 | 28.6 | 10.7 | 34.2 | 7.4 | 0.83 |
| Dox1 | NaCl 0.9% | 98 | 12.3 | 0.34 | 29.9 | 11.3 | 32.6 | 7.2 | 0.91 |

Additional measurement conditions: constant cross flow XF=0.3 at detector flow DF=0.5 mL/min, injected mass 25 µg, long channel equipped with a 10 kDa RC and a 350 µm spacer, applying a focus time of 8 min, and a focus flow of 2 mL/min, sample diluted at 1 mg/mL in the elution buffer (PBS from Hyclone).

Table S15: Summary of results obtained for Dox1 and its control Dox1C applying a focus time of 3, 5 or 8 min. *R*_h_ measured by QELS (Wyatt) at the angle position 99°. Recovery, retention time and retention ratio were measured at an absorbance of 280 nm. The average and spread of *R*_g_ (Berry model) and *R*_h_ (single exponential fit) were determined across the FWHM of the peak. Only one injection for each condition was tested, except where specified. *Average (standard deviation) are reported for 5 replicate measurements.

| Sample | Focus time (min) | Recovery (%) | Retention  time (min) | Retention ratio | *R*_g_ (nm) | *R*_g_ Spread (nm) | *R*_h_ (nm) | *R*_h_ Spread (nm) | *R*_g_/*R*_h_ |
| --- | --- | --- | --- | --- | --- | --- | --- | --- | --- |
| Dox1 | 3 | 101 | 12.27 | 0.039 | 28.7 | 12.2 | 33.5 | 9.0 | 0.86 |
| Dox1 | 5 | 101 | 12.24 | 0.04 | 26.6 | 12.9 | 33.4 | 8.7 | 0.79 |
| Dox1 | 8* | 98 (1) | 11.9 (0.3) | 0.04 (0.03) | 28.9 (0.3) | 11.9 (0.6) | 34.2 (0.4) | 11.0 (1.4) | 0.84 (0.02) |
| Dox1C | 3 | 110 | 12.88 | 0.035 | 27.91 | 13.9 | 34.3 | 9.7 | 0.81 |
| Dox1C | 5 | 110 | 12.84 | 0.03 | 27.89 | 12.9 | 34.0 | 10.4 | 0.82 |
| Dox1C | 8 | 100 | 12.76 | 0.03 | 28.66 | 13.11 | 34.0 | 10.9 | 0.84 |

Additional measurement conditions: constant cross flow XF=0.3 at detector flow DF=0.5 mL/min, injected mass 25 µg, long channel equipped with a 10 kDa RC membrane and a 350 µm spacer, applying a focus flow of 2 mL/min, sample diluted to 1 mg/mL in the elution buffer (PBS from Hyclone).

Table S16: Summary of results for Dox2 and Dox4, applying a focus time of 3, 5 or 8 min. *R*_h_ was measured by QELS (Wyatt) at the angle position 99°. Recovery, retention time and retention ratio were measured at an absorbance of 280 nm. The average and spread of *R*_g_ (Berry model) and *R*_h_ (single exponential fit) were determined across the FWHM of the peak. Only one injection for each condition was tested, except where specified. *In the case of the Dox2, which is composed of multiple populations, the size value calculated at the LS 90° peak maximum are also reported.

| Sample | Focus  (min) | Replicates | | Recovery (%) | Retention  time (min) | Retention  ratio | *R*_g_ (nm) | *R*_g_ Spread (nm) | *R*_h_ (nm) | *R*_h_ Spread (nm) | *R*_g_/*R*_h_ |
| --- | --- | --- | --- | --- | --- | --- | --- | --- | --- | --- | --- |
| Dox4 | 3 | 1 | 98 | | 11.3 | 0.030 | 30.0 | 10.5 | 35.1 | 12.0 | 0.85 |
| Dox4 | 5 | 1 | 99 | | 11.1 | 0.032 | 29.76 | 10.6 | 35.4 | 8.4 | 0.84 |
| Dox4 | 8 | 4 | 101 (1) | | 11.1 (0.06) | 0.032 (0.001) | 30.0 (0.16) | 10.9 (0.1) | 35.0 (0.2) | 10.3 (0.7) | 0.85 (0.01) |
| Dox2 | 3 | 3 | 84 (5) | | Peak 1: 8.4 (0.04)  Peak 2: 14.5 (0.3) | Peak 1: 0.041 (0.004)  Peak 2: 0.023 (0.001) | Peak 1*: 20.0 (0.6)  Peak 2*: 35.9 (0.4)  FWHM: 45 (2) | FWHM: 60 (4) | Peak 1*: 27 (1)  Peak 2*: 38.1 (0.6)  FWHM: 61 (6) | FWHM: 76 (8) | Peak 1*: 0.75 (0.04)  Peak 2*: 0.94 (0.07)  FWHM: 0.74 (0.04) |
| Dox2 | 5 | 3 | 96 (5) | | Peak 1: 9.2 (1.2)  Peak 2: 14.5 (0.3) | Peak 1: 0.039 (0.004)  Peak 2: 0.025 (0.004) | Peak 1*: 18.8 (0.2)  Peak 2*: 35.3 (0.4)  FWHM: 49 (1) | FWHM: 66 (3) | Peak 1*: 25.4 (0.3)  Peak 2*: 37.7 (0.8)  FWHM: 62 (4) | FWHM: 81 (27) | Peak 1*: 0.77 (0.01)  Peak 2*: 0.93 (0.02)  FWHM: 0.79 (0.03) |
| Dox2 | 8 | 5 | 93 (6) | | Peak 1: 8.6 (0.2)  Peak 2: 15.0 (0.7) | Peak 1: 0.048 (0.008)  Peak 2: 0.027 (0.005) | Peak 1: 20 (2)  Peak 2: 35.5 (0.2)  FWHM: 46 (1) | FWHM: 61 (1) | Peak 1: 26 (2)  Peak 2: 39 (1)  FWHM: 60 (6) | FWHM: 76 (16) | Peak 1: 0.76 (0.03)  Peak 2: 0.90 (0.03)  FWHM: 0.72 (0.05) |

Additional measurement conditions: constant cross flow XF=0.3 at detector flow DF=0.5 mL/min, injected mass 25 µg, long channel equipped with a 10 kDa RC membrane and a 350 µm spacer, applying a focus flow of 2 mL/min, sample diluted to 1 mg/mL in the elution buffer (PBS from Hyclone).

Table S17: Concentration series on Dox1. Summary of results for Dox1 in the (5 to 200) µg range. *R*_h_ was measured by QELS (Wyatt) at the angle position 99°. Recovery, retention time and retention ratio were measured at an absorbance of 280 nm. The average and spread of *R*_g_ (Berry model) and *R*_h_ (single exponential fit) were determined across the FWHM of the peak. Only one injection for each condition was tested, except where specified. *Average (standard deviation) for 5 replicate measurements.

| Sample | Mass injected (µg) | Recovery (%) | Retention  time (min) | Retention  ratio | *R*_g_ (nm) | *R*_g_ Spread (nm) | *R*_h_ (nm) | *R*_h_ Spread (nm) | *R*_g_/*R*_h_ |
| --- | --- | --- | --- | --- | --- | --- | --- | --- | --- |
| Dox1 | 5 | na | 13.20 | 0.04 | 32.48 | 6.9 | 47.1 | 4.4 | 0.69 |
| Dox1 | 15 | na | 12.30 | 0.04 | 22.90 | 12.0 | 35.4 | 9.9 | 0.64 |
| Dox1 | 25* | 98 (1) | 11.9 (0.3) | 0.04 (0.03) | 28.9 (0.3) | 11.9 (0.6) | 34.2 (0.4) | 11.0 (1.4) | 0.84 (0.02) |
| Dox1 | 50 | na | 12.73 | 0.04 | 28.56 | 12.2 | 33.5 | 9.3 | 0.85 |
| Dox1 | 100 | na | 12.04 | 0.03 | 28.53 | 12.2 | 32.4 | 9.1 | 0.88 |
| Dox1 | 200 | na | 12.69 | 0.03 | 29.53 | 12.6 | 30.8 | 8.5 | 0.95 |

Additional measurement conditions: constant cross flow XF=0.3 at detector flow DF=0.5 mL/min, long channel equipped with a 10 kDa RC membrane and a 350 µm spacer, applying a focus flow of 2 mL/min for 8 min, sample diluted to 1 mg/mL in the elution buffer (PBS from Hyclone).

Table S18: Summary of results for Dox2 in the (15 to 200) µg injected mass range. *R*_h_ was measured by QELS (Wyatt) at position LS-12. Recovery, retention time and retention ratio were measured at an absorbance of 280 nm. The average and spread of *R*_g_ (Berry model) and *R*_h_ (single exponential fit) were determined across the FWHM of the peak. Only one injection for each condition was tested, except where specified. In the case of Dox2, which is composed of multiple populations, the size value calculated at the LS 90° peak maximum are also reported. *Average (standard deviation) for 5 replicate measurements.

| Sample | Mass injected (µg) | Recovery (%) | Retention time (min) | Retention ratio | *R*_g_ (nm) | *R*_g_ Spread (nm) | *R*_h_ (nm) | *R*_h_ Spread (nm) | *R*_g_/*R*_h_ |
| --- | --- | --- | --- | --- | --- | --- | --- | --- | --- |
| Dox2 | 15 | 63 | Peak 1: 9.1  Peak 2: 13.8 | Peak 1: 0.07  Peak 2: 0.04 | Peak 1: 21.9  Peak 2: 33.8 | FWHM: 28 | Peak 1: 26.8  Peak 2: 41.0  FWHM: na | FWHM: 38 | Peak 1: 0.81  Peak 2: 0.82 |
| Dox2 | 25* | 93 (6) | Peak 1:  8.6 (0.2)  Peak 2: 15.0 (0.7) | Peak 1: 0.048 (0.008)  Peak 2: 0.027 (0.005) | Peak 1:  20 (2)  Peak 2: 35.5 (0.2) | FWHM: 61 (1) | Peak 1:  26 (2)  Peak 2: 39 (1) | FWHM: 56 (16) | Peak 1: 0.76 (0.03)  Peak 2: 0.90 (0.03) |
| Dox2 | 50 | 72 | Peak 1: 9.4  Peak 2: 15.6 | Peak1: 0.04  Peak 2: 0.024 | Peak 1: 21.0  Peak 2: 35.3 | FWHM: 45 | Peak 1: 27.7  Peak 2: 40.0  FWHM: 52 | FWHM: 36 | Peak 1: 0.76  Peak 2: 0.88 |
| Dox2 | 100 | 73 | Peak 1: 8.4  Peak 2: 14.3 | Peak 1: 0.04  Peak 2: 0.03 | Peak 1: 18.0  Peak 2: 37.0 | FWHM: 45 | Peak 1: 24.0  Peak 2: 40.5 | FWHM: 40 | Peak 1: 0.77  Peak 2: 0.91 |
| Dox2 | 200 | 90 | Peak 1: 8.7  Peak 2: 16.3 | Peak 1: 0.04  Peak 2: 0.02 | Peak 1: 19.7  Peak 2: 40.1 | FWHM: 60 | Peak 1: 24.2  Peak 2: 40.0 | FWHM: 56 | Peak 1: 0.81  Peak 2: 1.01 |

Additional measurement conditions: constant cross flow XF=0.3 at detector flow DF=0.5 mL/min, long channel equipped with a 10 kDa RC membrane and a 350 µm spacer, applying a focus flow of 2 mL/min for 8 min, sample diluted to 1 mg/mL in the elution buffer (PBS from Hyclone).

Table S19: Comparison of results for multiple liposomal formulations using the optimized AF4 method. *R*_h_ was measured by QELS (Wyatt) at position LS-12. Recovery, retention time and retention ratio were measured at an absorbance of 280 nm. The average and spread of *R*_g_ (Berry model) and *R*_h_ (single exponential fit) were determined across the FWHM of the peak. The mean (COV) of at least 3 replicate injections is reported for each parameter.

| Sample | Replicates | Recovery (%) | Retention time (min) | *R*_g_ (nm) | *R*_g_ Spread (nm) | *R*_h_ (nm) | *R*_h_ Spread (nm) | *R*_g_/*R*_h_ |
| --- | --- | --- | --- | --- | --- | --- | --- | --- |
| Dox1 | 5 | 98 (1) | 11.9 (2.5%) | 28.9 (1%) | 11.9 (5%) | 34.2 (1%) | 11.0 (12%) | 0.84 (2%) |
| Dox1C | 3 | 92 (0.4) | 13.2 (0.2%) | 32.1 (0.1%) | 11.9 (1%) | 36.3 (0.1%) | 10.8 (1%) | 0.88 (1%) |
| Dox3 | 3 | 102 (3) | 12.6 (0.2%) | 30.7 (1.3%) | 10.0 (1%) | 35.5 (0.3%) | 10 (10%) | 0.86 (1%) |
| Dox4 | 4 | 101 (1) | 11.1 (1%) | 30.0 (0.5%) | 10.9 (1%) | 35.0 (0.6%) | 10.3 (7%) | 0.84  (1%) |
| Dox2 | 5 | 93 (6) | Peak 1: 8.6 (2.3%)  Peak 2: 15.0 (5%) | Peak 1:  20 (10%)  Peak 2: 35.5 (0.6%)  FWHM: 46 (2.2%) | FWHM:  61 (1.6%) | Peak 1:  26 (8%)  Peak 2: 39 (2.6%)  FWHM: 60 (10%) | FWHM:  76 (21%) | Peak 1: 0.76 (4%)  Peak 2: 0.90 (3%)  FWHM: 0.72 (7%) |
| Cipro | 3 | 91 (6) | 13.5 (0.4%) | 32.7 (1.5%) | 10.8 (3%) | 35.1 (1%) | 8 (12%) | 0.932 (0.6%) |

*Only for Dox2, the size value calculated at LS 90° peak maxima are also reported.

Table S20: Summary of results obtained for Dox1 and Dox2 (1 mg/mL with or without 10 % FBS), in a long channel (Wyatt) and applying the optimized method. *R*_h_ was measured by QELS (Wyatt) at position LS-12. Recovery, retention time and retention ratio were measured at an absorbance of 280 nm. The average and spread of *R*_g_ (Berry model) and *R*_h_ (single exponential fit) were determined across the FWHM of the peak.

| Sample | Replicates | Recovery (%) | Retention  time (min) | Retention  ratio | *R*_g_ (nm) | *R*_g_ Spread (nm) | *R*_h_ (nm) | *R*_h_ Spread (nm) | *R*_g_/*R*_h_ |
| --- | --- | --- | --- | --- | --- | --- | --- | --- | --- |
| Dox1 | 5 | 98 (1) | 11.9 (0.3) | 0.04 (0.03) | 28.9 (0.3) | 11.9 (0.6) | 34.2 (0.4) | 11.0 (1.4) | 0.84 (0.02) |
| Dox1+FBS 10% | 1 | 101 | 11.02 | 0.032 | 29.3 | 11.7 | 34.0 | 9 | 0.861 |
| Dox2 | 5 | 93 (6) | Peak 1:  8.6 (0.2)  Peak 2: 15.0 (0.7) | Peak 1: 0.048 (0.008)  Peak 2: 0.027 (0.005) | Peak 1:  20 (2)  Peak 2: 35.5 (0.2) | FWHM:  61 (1) | Peak 1:  26 (2)  Peak 2: 39 (1) | FWHM: 56 (16) | Peak 1: 0.76 (0.03)  Peak 2: 0.90 (0.03) |
| Dox2+ FBS 10% | 1 | 101 | Peak 1: 8.85  Peak 2: 14.5 | Peak 1: 0.062  Peak 2: 0.038 | Peak 1: 18.9  Peak 2: 37.3 | FWHM: 55 | Peak 1: 29.2  Peak 2: 39.7 | FWHM: 70 | Peak 1: 0.65  Peak 2: 0.93 |

1. The identification of any commercial product or trade name does not imply endorsement or recommendation by the National Institute of Standards and Technology. [↑](#footnote-ref-1)
2. pH was measured using InLab Semi-Micro Combination pH electrode and model Mettler Toledo pH meter after calibration with NIST Traceable buffers at ambient temperature. [↑](#footnote-ref-2)
